# Neutrophil Extracellular Traps Promote Inflammatory Responses in Psoriasis via Activating Epidermal TLR4/IL-36R Crosstalk

**DOI:** 10.3389/fimmu.2019.00746

**Published:** 2019-04-05

**Authors:** Shuai Shao, Hui Fang, Erle Dang, Ke Xue, Jieyu Zhang, Bing Li, Hongjiang Qiao, Tianyu Cao, Yuchen Zhuang, Shengxian Shen, Tongmei Zhang, Pei Qiao, Caixia Li, Johann E. Gudjonsson, Gang Wang

**Affiliations:** ^1^Department of Dermatology, Xijing Hospital, Fourth Military Medical University, Xi'an, China; ^2^Department of Dermatology, University of Michigan, Ann Arbor, MI, United States

**Keywords:** psoriasis, neutrophil extracellular traps, IL-36, TLR4, keratinocyte

## Abstract

Epidermal infiltration of neutrophils is a hallmark of psoriasis, where their activation leads to release of neutrophil extracellular traps (NETs). The contribution of NETs to psoriasis pathogenesis has been unclear, but here we demonstrate that NETs drive inflammatory responses in skin through activation of epidermal TLR4/IL-36R crosstalk. This activation is dependent upon NETs formation and integrity, as targeting NETs with DNase I or CI-amidine *in vivo* improves disease in the imiquimod (IMQ)-induced psoriasis-like mouse model, decreasing IL-17A, lipocalin2 (LCN2), and IL-36G expression. Proinflammatory activity of NETs, and LCN2 induction, is dependent upon activation of TLR4/IL-36R crosstalk and MyD88/nuclear factor-kappa B (NF-κB) down-stream signaling, but independent of TLR7 or TLR9. Notably, both TLR4 inhibition and LCN2 neutralization alleviate psoriasis-like inflammation and NETs formation in both the IMQ model and K14-VEGF transgenic mice. In summary, these results outline the mechanisms for the proinflammatory activity of NETs in skin and identify NETs/TLR4 as novel therapeutic targets in psoriasis.

## Introduction

Psoriasis is a chronic recurrent inflammatory skin disease with a complex pathogenesis (1, 2). It is generally considered that the main pathogenic driver of psoriasis is an intricate interplay between infiltrated inflammatory cells and activated keratinocytes (3, 4). While the roles of dendritic cells (DCs), T cells, and macrophages have been extensively investigated (5–8), the contribution of neutrophils, the most abundant leukocyte population of the immune system in humans, has remained unclear.

Neutrophils play a crucial role in a wide variety of conditions, including infections, autoimmune, neoplastic, and chronic inflammatory diseases (9–12). Presence of neutrophils is one of the hallmark histologic features of psoriasis, with neutrophils located both in the dermis and within the epidermis where they form small microabscesses as is seen in chronic plaque psoriasis, or larger intraepidermal neutrophilic collections characteristic of pustular psoriasis (2). No studies have comprehensively addressed the role of neutrophils in psoriasis, but neutrophil involvement is suggested by the results from several clinical studies, in which selective depletion of granulocytes and monocytes by adsorptive apheresis led to marked improvement in psoriasis symptoms (13, 14).

Neutrophils are very efficient phagocytes and this is central to their microbicidal role (15). In addition, neutrophils can be a major source of antimicrobial proteins (AMPs) such as S100A8/S100A9, cathelicidin (LL37) and lipocalin2 (LCN2), all of which can participate in and amplify inflammatory responses (16–19). Neutrophils may also undergo a cell death process called NETosis upon activation, in which they extrude their nuclear material into the extracellular space (20, 21). These structures called neutrophil extracellular traps (NETs) are large, web-like structures composed of decondensed DNA, histones, and granule proteins (22), and their presence has been described in psoriatic skin, where they may act by inducing increased expression of human β-defensin-2 (23). In short, neutrophils and NETs can contribute to inflammation through several different mechanisms, through inflammasome activation (24), triggering of TLR7 and TLR9 by self-antigen complexes such as LL37-DNA (25), promoting macrophage pyroptosis (26), or through processing and activation of IL-36 cytokines (27, 28).

Based on these considerations, we explored the mechanisms by which neutrophils and NETs affect the pathogenesis of psoriasis. We confirm that psoriatic neutrophils are activated and form NETs in psoriasis patients compared to healthy controls. Using both *in vivo* and *in vitro* approaches, we demonstrate that neutrophils, through release of NETs, amplify skin inflammation through activation of IL-36 and toll-like receptor 4 (TLR4) signaling. Furthermore, IL-36 and TLR4 signaling act synergistically to induce expression of the neutrophil chemoattractant LCN2 in keratinocytes, further increasing neutrophil infiltration into the skin, and thereby amplifying the inflammatory cascade. These results demonstrate a major role for neutrophils and NETs in amplifying skin inflammation and identify NETs/TLR4 as novel therapeutic targets in psoriasis.

## Materials and Methods

### Patients and Samples

All analyses of human materials were performed in full agreement with institutional guidelines, with the approval of the Ethical committee of the Fourth Military Medical University (reference number KY20173053-1), and conducted according to the principles in the Declaration of Helsinki. Informed consent to collect blood and skin lesions were obtained from all subjects enrolled in the study.

We chose psoriasis patients (twenty-five men and seventeen women, age ranged from 18 to 59 years with mean of 35.6 years old) who visited our Department at Xijing Hospital without any other systemic diseases or any systemic treatment for a minimum of 6 weeks. Normal control blood samples were collected from age- and sex- matched healthy volunteers working at our Department (sixteen men and twenty-two women, age ranged from 25 to 42 years with mean of 30.4 years old). Control skin biopsies were obtained from discarded healthy skin from donors (one men and three women, age ranged from 21 to 45 years with mean of 29.1 years old) who were admitted to the Department of Plastic Surgery at Xijing Hospital.

### Microarray Data Processing and Analysis

Total RNA containing small RNA was extracted from peripheral neutrophils of psoriasis patients and healthy controls (*n* = 3 for each group) by using the trizol reagent (Invitrogen). The Affymetrix PrimeView Human Gene Expression Array was used in this study and performed by CapitalBio Corporation (Beijing, China). The scanned images were assessed and analyzed to generate raw data files saved as CEL files using Affymetrix GeneChip Operating software (GCOS 1.4). The quality of each CEL file was assessed using Affymetrix Expression Console Software according to the Affymetrix standard protocol. And the increase/decrease of gene expression in neutrophils from psoriasis patients compared with those from healthy controls was calculated as a log2-fold change to obtain a symmetric distribution around zero. For functional clustering, genes were annotated with Gene Ontologies (www.geneontology.org/).

### Mouse Experiments

The animal studies were approved by the institutional review board, and carried out in accordance with the National Institutes of Health guide for the care and use of Laboratory animals. Female BALB/c mice aged 6 to 8 weeks used for these studies were obtained from the Department of Laboratory Animal Medicine of the Fourth Military Medical University and bred and housed individually in a specific pathogen-free barrier facility. Mice were randomly assigned to groups of three mice each, and received Cl-amidine (10 mg/kg/d, 10599; Cayman Chemical) or DNase I (10 mg/kg/d, 18068-015; Invitrogen) or an equal volume of PBS by daily intravenous injection (29, 30) 4 h prior to imiquimod (IMQ) induction. The mice then received a daily topical dose of 6.25 mg IMQ cream (INova Pharmaceuticals) or Vaseline on the shaved back for consecutive 7 days.

To investigate the role of TLR4 signaling in psoriasis, we used cream base to blend the siRNA targeting TLR4 (0.5 nmol in 1 mg emulsion matrix/ear, Ribobio) or TLR4 inhibitor TAK-242 (0.5 mg in 1 mg emulsion matrix/ear, A3850; APExBIO). For each mouse, the ears were applied with TLR4 siRNA, control (NC) siRNA, TAK-242 or DMSO mixed with emulsion matrix every morning, and the right ears were injected with murine PMA NETs (isolated from PMA treated murine neutrophils; 25μg/ear) subcutaneously every other day and the left with same volume of culture medium from unstimulated murine neutrophils. Both ears were also treated with 4 mg IMQ or Vaseline daily for continuative 7 days.

To evaluate the pathogenic role of LCN2 in psoriasis, anti-LCN2 mAb (100 μg/mouse, MAB1857; R&D Systems) or rat IgG2a isotype control antibody (100 μg/mouse, MAB006; R&D Systems) were intraperitoneally (i.p.) injected 4 h prior the first IMQ induction and every 48 h thereafter. Shaved mouse dorsal skin was treated daily with IMQ cream or Vaseline for 7 days. In addition, K14-VEGF mice were provided by the Department of Pharmacy in Changhai Hospital, and were i.p. injected with anti-LCN2 mAb or isotype control antibody every 48 h for consecutive 14 days.

Blood was collected by the tail-vein approach on day 7, and serum was stored at −80°C for later ELISA.

### MPO-DNA Enzyme-Linked Immunosorbent Assay (ELISA)

To quantify NETs in human serum, we employed a capture ELISA based on MPO associated with DNA. For the capture antibody, 5 μg/ml anti-MPO mAb (07-496; Upstate) was coated onto 96-well-plates at 4°C overnight. After washing 3 times (300 μl each), 50 μl of serum samples was added to the wells with 50 μl incubation buffer containing a peroxidase-labeled anti-DNA mAb in Cell Death ELISAPLUS kit (1:25, Roche). The plate was incubated for 4 h, shaking at 300 rpm at room temperature. After 3 washes (300 μl each), 100 μl peroxidase substrate (ABTS) was added to incubate at room temperature in the dark for 20 min. Then absorbance at 405 nm wavelength was measured.

### NETs Generation

2 × 10^6^ neutrophils were seeded in 12-well-plates in serum-free Roswell Park Memorial Institute (RPMI) (Gibco) and were then stimulated to release NETs by phorbol 12-myristate 13-acetate (PMA) (50nM, P1585; Sigma) for 4 h at 37°C. Afterwards, the medium was carefully removed and the cell layer were washed softly with 2 ml PBS without Ca and Mg. Then the washing PBS was collected after vigorous agitation and centrifuged for 10 min at 450 g at 4°C and cell-free NETs structures were collected in supernatant phase.

### Mass Spectrum of Contents of NETs

For NETs protein isolation, isolated 2 × 10^6^ neutrophils were seeded in 12-well-plates in serum-free RPMI (Gibco) and were then stimulated to release NETs by PMA (50 nM, Sigma) for 4 h at 37°C. Supernatant was removed and each well was washed carefully with RPMI. NETs were then digested with 10U/ml DNase I (Invitrogen) for 20 min, and ethylenediaminetetraacetic acid (EDTA; 5 mM, Invitrogen) was used to block the activity of DNase I. Samples were then centrifuged at 300 g to remove whole cells and 16,000 g to remove debris. Then 85 μg of proteins of PMA-induced NETs from psoriatic neutrophils were identified using Nano-LC/MALDI-MS (Capitalbio Technology Corporation).

### Primary Human Keratinocytes Culture and Stimulation

Foreskin keratinocytes were obtained from anonymized donors. The study was approved by the ethics committee of the Fourth Military Medical University and conducted according to the principles of Declaration of Helsinki. Informed written consent was given by every patient or their parents. In short, skin specimens were cleaned of adipose tissue, cut into small fragments and then incubated in Dispase II (D4693; Sigma) at 37°C for 30 min. The epidermal layer containing keratinocytes was carefully separated from the dermis using fine forceps, and 0.25% trypsin–EDTA (Gibco) was added for 12 min at 37°C to release epidermal keratinocytes. The trypsin reactivity was neutralized by addition of 10% FBS. Following centrifugation (190 g for 10min), the cell pellet was collected.

Then keratinocytes were cultured in a serum-free Keratinocyte-SFM Media Kit (containing 0.125 μg/ml epidermal growth factor) (Gibco) in a humidified 5% CO_2_ incubator, and passage 3 to 4 cells were used for experiments. In short, cells at 50 to 70% confluency were stimulated for different time periods with NETs (1 μg/ml, 5 μg/ml, 10 μg/ml), recombinant Histone3 (5 μg/ml, ab132921; Abcam), S100A8 (10 μg/ml, 11138-H08B; Sino Biological), S100A9 (10 μg/ml, 11145-H08B; Sino Biological), HSP70 (10 μg/ml, 11660-H07H; Sino Biological), LCN2 (10 μg/ml, ab175463; Abcam). To inhibit PAD4 activity, neutrophils were pretreated with Cl-amidine (100 μM; Cayman, MI, USA) for 15 min. In another experiment of cell stimulation, IL-36γ (100 ng/ml, 10124-HNCE1; Sino Biological) and IL1β (50 ng/ml, GMP-10139-HNAE; Sino Biological), IL36R (2 μg/mL, MAB8721; R&D), and IL1RI antibodies (2 μg/mL, AF269; R&D) were also used.

### Statistical Analysis

For statistical analysis, data obtained from at least three independent experiments were performed using GraphPad Prism software versions 6 (GraphPad software, San Diego, CA, USA). Statistical significance was determined using Student's unpaired two-tailed *t* test or analysis of variance (ANOVA) as indicated in the legend (^*^P < 0.05, ^**^P < 0.01, ^***^P < 0.001, ^****^P < 0.0001). Flow cytometry data was analyzed using FlowJo v10. The number of sampled units, n, is indicated in the Figure legends.

The other methods can be found in Supplemental Materials and methods. The information of primers used in this study was listed in Table S2.

## Results

### Psoriatic Neutrophils Are Pre-activated and Form NETs

**Figure 1:**
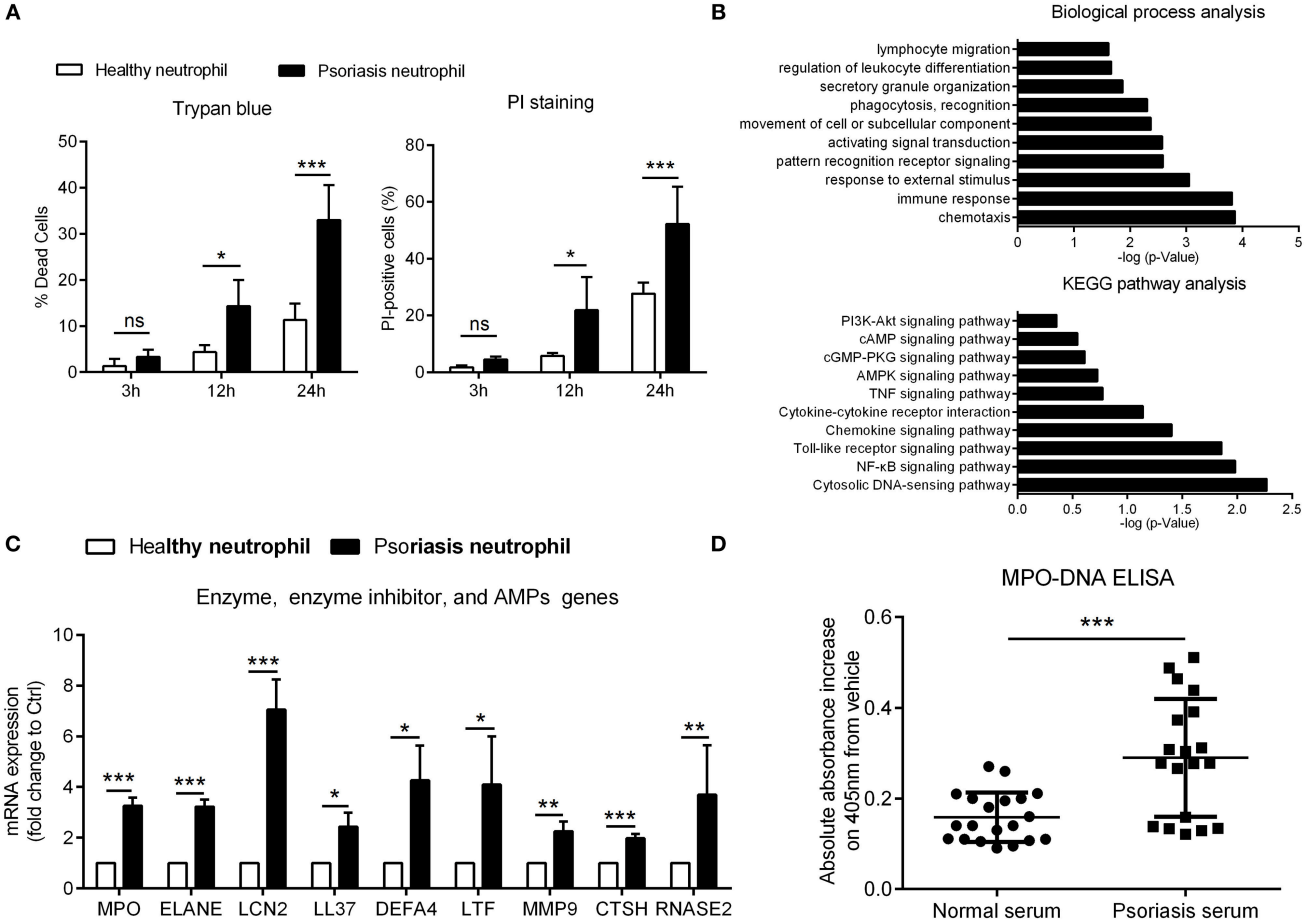
Psoriatic neutrophils are pre-activated and form NETs **(A)** Cell viability of neutrophils from psoriasis patients and normal controls was evaluated at different time points using trypan blue and AnnexinV-PI. Two-way ANOVA, *n* = 3 (mean±SD). (**B)** DEGs in neutrophils of psoriasis patients (*n* = 3) compared to healthy controls (*n* = 3) were assigned to gene categories according to their biological functions and involved pathways. **(C)** Confirmation of enhanced mRNA expressions in psoriasis neutrophils compared with healthy neutrophils. Two-tailed Student's *t*-test, *n* = 3 per group (mean±SD). (**D)** The MPO-DNA complex level in serum from psoriasis patients and normal controls. *n* = 20, each data point represents an individual. Two-tailed Student's *t*-test. *P < 0.05, **P < 0.01, ***P < 0.001, ns, not significant, ***P < 0.001. All the bars represent the average of three independent experiments. ELANE, elastase; DEFA4, defensin α4; LTF, lactotransferrin; MMP9, matrix metallopeptidase 9; CTSH, cathepsin H; RNASE2, ribonuclease A family member 2.

To explore neutrophil participation in psoriasis pathogenesis, we compared cell viability of neutrophils in psoriasis patients against healthy controls. Psoriasis neutrophils underwent spontaneous death at a higher rate than healthy neutrophils *in vitro* (Figure 1A; Figure S1A). To understand the underlying cause of this accelerated death and identify additional differences between psoriasis neutrophils and healthy ones, we analyzed microarray gene expression data from peripheral neutrophils from three psoriasis patients and three healthy controls (GEO: GSE106087). Using Gene ontology analysis, the differentially expressed genes (DEGs) in psoriasis vs. healthy neutrophils could be assigned to various inflammatory functions, including “chemotaxis,” “response to stimulus,” and “leukocyte activation,” indicating that circulating neutrophils are pre-activated in psoriasis patients. KEGG (Kyoto Encyclopedia of Genes and Genomes) pathways analysis demonstrated the enrichment for “cytosolic DNA-sensing pathway,” “NF-κB signaling pathway,” and “Toll-like receptor signaling pathway” (Figure 1B). Moreover, the top upregulated genes were verified by quantitative real-time polymerase chain reaction (qRT-PCR) (Figure 1C). These increased DEGs found in psoriasis neutrophils were genes encoding for enzymes, proteases, and cathelicidin which have been shown to be indispensable components and inducers of NETs formation (22, 31). Therefore, we explored the presence of NETs in psoriasis patients by quantification of circulating MPO-DNA complexes, which showed significant increase in serum of psoriasis patients compared to healthy individuals (Figure 1D). Using cell immunofluorescence, we demonstrated that interleukin (IL)-17A, tumor necrosis factor (TNF)-α, high mobility group box-1 (HMGB1), and LCN2, all of which are elevated in psoriasis (4, 19, 32), were potent inducers of NETs formation, and comparable to induction by phorbol 12-myristate 13-acetate (PMA), which was used as a positive control (Figure S1B). These data demonstrate that neutrophils are pre-activated and primed to form NETs in psoriasis patients.

### Targeting NETs Attenuates Psoriasis-Like Inflammation *in vivo*

**Figure 2:**
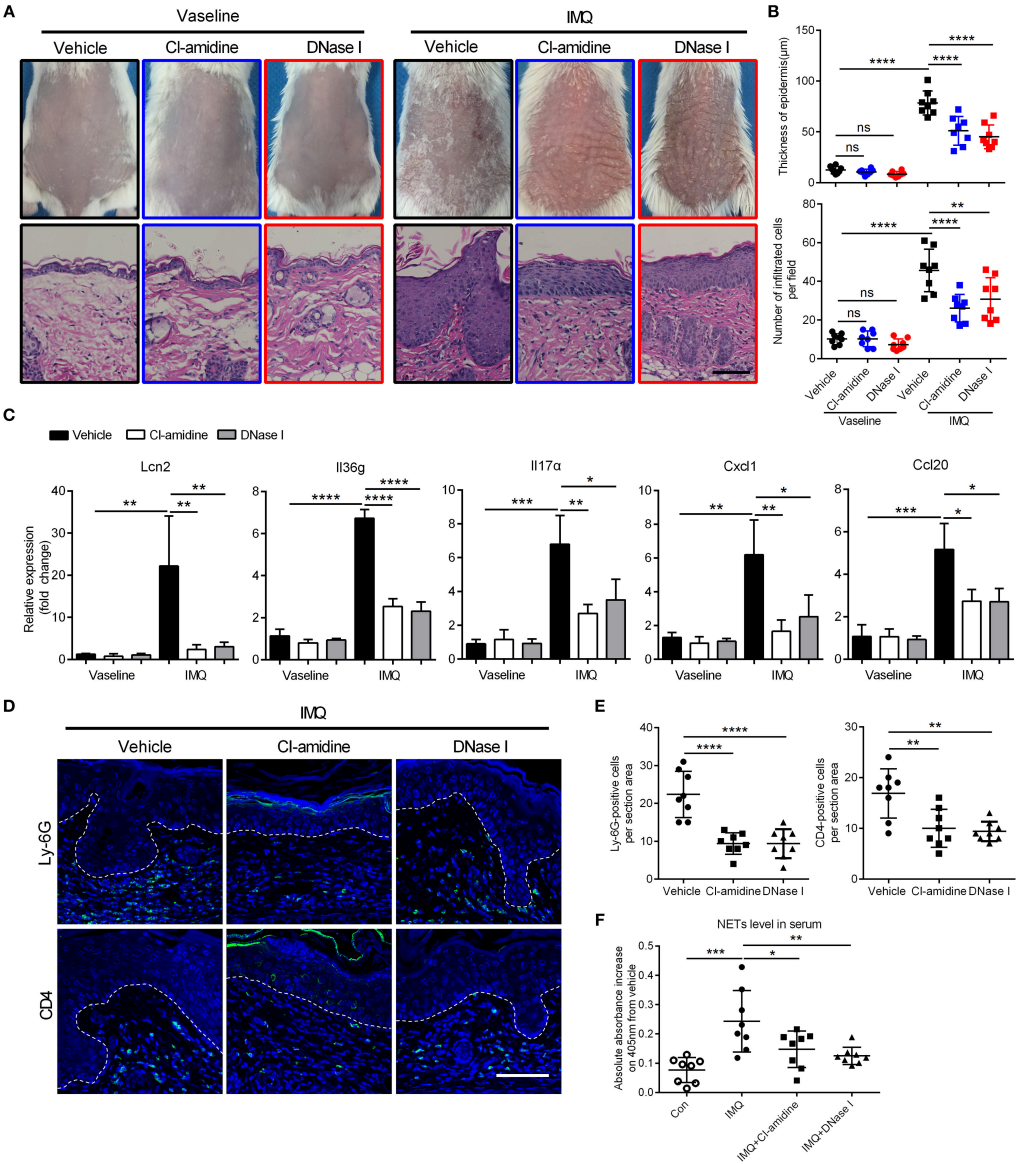
Targeting NETs attenuates psoriasis-like inflammation *in vivo* **(A)** The phenotype and H&E staining of back skin from CI-amidine, DNase I, or PBS-administrated mice with IMQ or Vaseline treatment, three mice per group. Scale bars, 100 μm. (**B)** Quantification of epidermal thickening and infiltrated immunocytes according to H&E staining in a One-way ANOVA, *n* = 8 per group (mean±SD). **(C)** QRT-PCR analyses of psoriasis-related cytokines and molecules from skin samples of mice described as in A One-way ANOVA, *n* = 8 per group (mean±SD). **(D, E)** Representative immunofluorescence and quantification of skin sections from CI-amidine, DNase I, or PBS-treated IMQ mice for neutrophil marker Ly-6G and T cell marker CD4. Scale bars, 100 μm. One-way ANOVA, *n* = 8 per group (mean±SD). **(F)** The MPO-DNA complex quantification indicating NETs level in serum from psoriasis patients and normal controls. *n* = 8, each data point represents an individual. One-way ANOVA. *P < 0.05, **P < 0.01, ***P < 0.001, ****P < 0.0001. All the bars represent the average of three independent experiments.

Similar to what was seen in human psoriasis (23), NETs were also detected in the skin lesions of IMQ mice (Figure S2A). To address the potential pathogenic role of NETs in this model, we treated IMQ mice with either CI-amidine, an inhibitor of peptidyl-arginine deiminase 4 (PAD4) that required in NETs formation (30), or DNase I to break down NETs. In the IMQ-induced mouse model, intravenous injection of CI-amidine or DNase I over a 7-day period resulted in decreased scaling, decreased acanthosis, and decreased inflammatory infiltrate, as observed by visual inspection and hematoxylin-eosin (H&E)-staining (Figures 2A,B). QRT-PCR analysis of inflamed back skin from IMQ mice revealed significantly lower expressions of Lcn2, Il36g, Il17α, Cxcl1, and Ccl20 in the CI-amidine- or DNase I-treated group compared to vehicle control (Figure 2C). Accordingly, the number of infiltrated T cells and neutrophils in the dermis were markedly decreased following CI-amidine or DNase I treatment (Figures 2D,E; Figure S2B). Importantly, the circulating MPO-DNA complexes indicating NETs level in serum was also reduced by CI-amidine or DNase I administration in IMQ mice (Figure 2F). Together, these data show that NETs can be targeted to treat psoriasis inflammation.

### NETs Activate Inflammatory Responses in Keratinocytes

**Figure 3:**
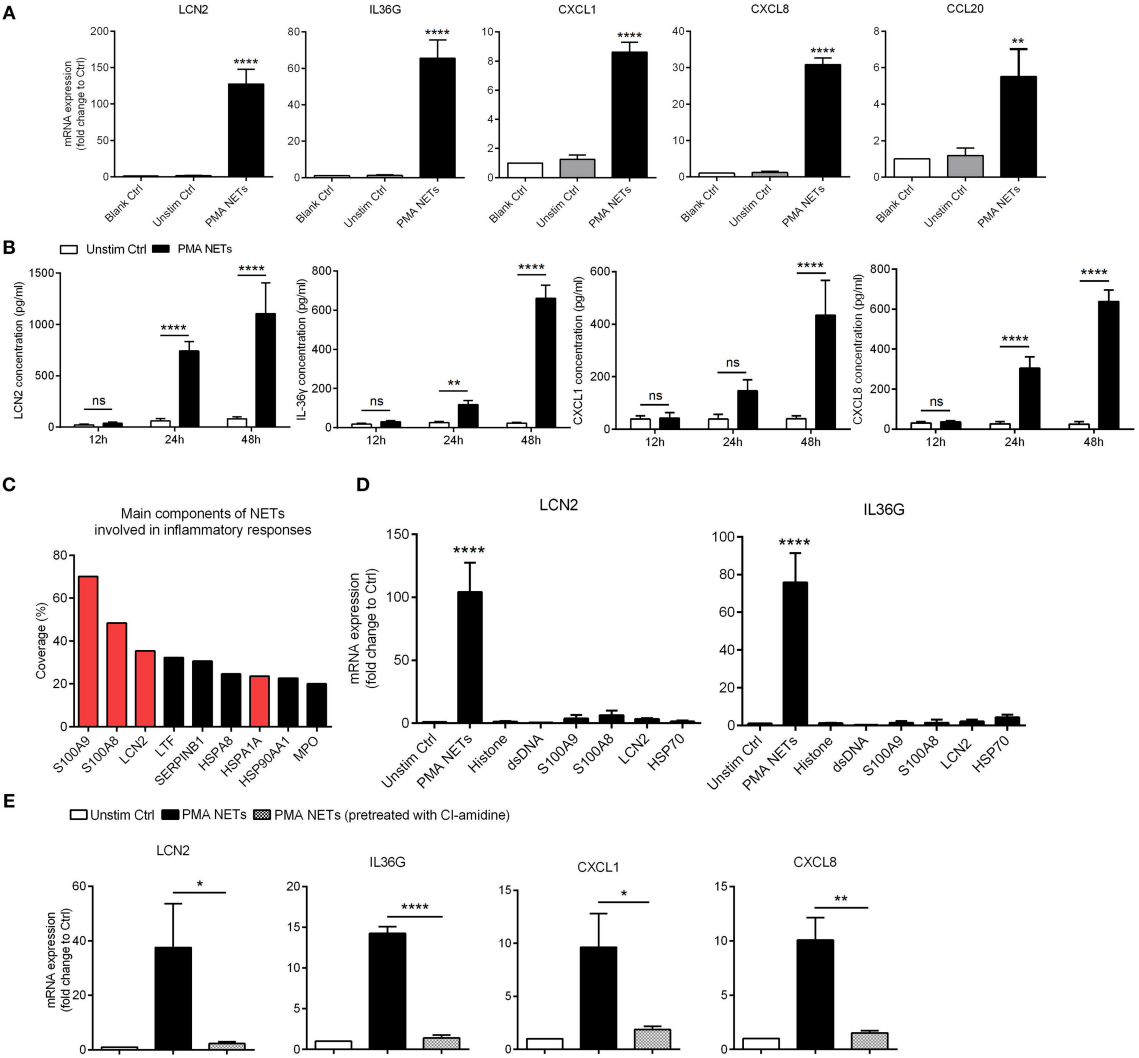
NETs activate inflammatory responses in keratinocytes **(A)** The mRNA expressions of inflammatory mediators in human primary keratinocytes stimulated with PMA-derived NETs or medium from unstimulated neutrophils for 24 h. One-way ANOVA. Data are expressed as means ± SD (human primary keratinocytes were obtained from three donors; peripheral blood samples were obtained from psoriasis patients). (**B)** The protein level of LCN2, CXCL8, CXCL1, and IL-36γ in the supernatant of cultured keratinocytes stimulated with NETs over indicated time. Data are expressed as means ± SD (*n* = 3). Two-way ANOVA. (**C)** Short summary of identified NETs-associated proteins that abundant in NETs supernatant that derived from neutrophils of psoriasis patients. **(D)** The mRNA expressions of LCN2 and IL36G in cultured keratinocytes stimulated with NETs or recombinant proteins over 24 h. One-way ANOVA (adjusted for Dunnett's test). Results represent means ± SD (*n* = 3). **(E)** The mRNA levels of LCN2, IL36G, CXCL1, and CXCL8 in keratinocytes stimulated by NETs or the supernatant from PMA-induced neutrophils that had been pre-treated with CI-amidine. One-way ANOVA. Values represent the means ± SD (*n* = 3). *P < 0.05, **P < 0.01, ****P < 0.0001. All the bars represent the average of three independent experiments. Unstim ctrl, unstimulated control.

As NETs are primarily located in close proximity to keratinocytes within psoriatic lesions (23), we wanted to determine whether NETs could directly activate keratinocytes to promote skin inflammation. To address this, we exposed human primary keratinocytes to purified PMA-induced NETs at different concentrations for 24 h, and compared against keratinocytes exposed to culture medium from unstimulated neutrophils (Figure S3A). NETs-induced genes in keratinocytes included LCN2, IL36G, and chemokines including CXCL8, CXCL1, and CCL20 (Figure 3A). In addition, NETs-stimulated keratinocytes released more LCN2, CXCL8, CXCL1, and IL-36γ protein into the culture medium than did control group (Figure 3B). Immunofluorescence showed marked expression of LCN2, CXCL8, CXCL1, and IL-36γ in psoriatic skin, but not in normal controls (Figure S3B).

We further analyzed the protein composition of PMA-induced NETs formed by psoriatic neutrophils using liquid chromatography followed by mass spectrometry (Table S1). Among them, S100A9, S100A8, LCN2, and HSP70 (HSPA1A) were amongst the most highly enriched proteins in NETs (Figure 3C). Considering that these molecules might independently contribute to keratinocyte activation, we treated keratinocytes with NETs and compared against keratinocytes treated with recombinant S100A9, S100A8, LCN2, and HSP70 proteins. QRT-PCR results confirmed our previous finding that NETs robustly up-regulated expression of LCN2, and IL36G, CXCL1, and CXCL8 in keratinocytes, whereas minimal responses were seen with each of the recombinant proteins (Figure 3D). Notably, when NETs formation was blocked by CI-amidine, their stimulatory effects on keratinocytes was significantly weakened (Figure 3E). Collectively, these data indicate that NETs work as an integrated unit to activate keratinocytes in psoriasis.

### TLR4/MyD88/TRAF6/TAK1 and NF-κB Signaling Mediate the NETs-Induced Immune Responses

**Figure 4:**
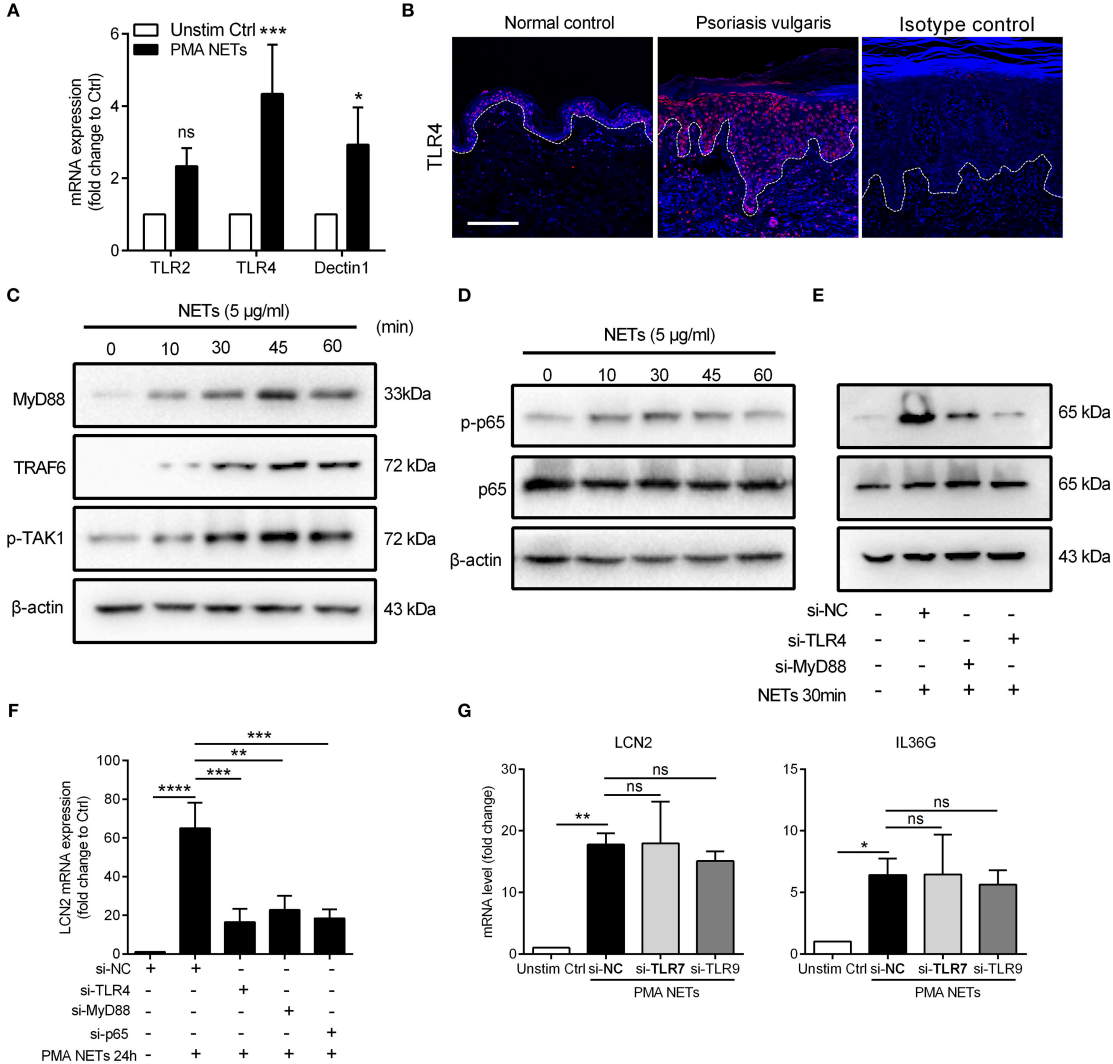
TLR4/MyD88/TRAF6/TAK1 and NF-κB signaling mediate the NETs-induced immune responses **(A)** QRT-PCR for TLR2, TLR4, and Dectin1 with cDNA from cultured keratinocytes stimulated with NETs. Data are expressed as means ± SD (*n* = 3). Two-way ANOVA. **(B)** Tissue immunofluorescence analysis of the location and expression of TLR4 in the skin lesions of psoriasis patients and normal controls. Scale bars, 100 μm. **(C)** The protein expressions of downstream adaptor molecules of TLR4 in NETs-stimulated keratinocytes. **(D)** The phosphorylation level of phospho-NF-κB p65 and total proteins at indicated time following NETs stimulation in primary keratinocytes. **(E)** The phosphorylation level of NF-κB p65 in TLR4- or MyD88-silenced keratinocytes with NETs stimulation for 30min. **(F)** LCN2 expression in TLR4-, MyD88-, or NF-κB p65-silenced keratinocytes with NETs stimulation. **(G)** The mRNA levels of LCN2 and IL36G in TLR7- or TLR9-silenced keratinocytes followed by NETs stimulation. One-way ANOVA, data are expressed as means ± SD (*n* = 3). *P < 0.05, **P < 0.01, ***P < 0.001, ****P < 0.0001, ns, not significant. All experiments were repeated for at least three times.

LCN2, the most highly expressed inflammatory mediator in NETs-treated keratinocytes, has been reported previously by our group to be abundant in the skin lesions of psoriasis patients and modulate neutrophil functions (19). Therefore, we chose LCN2 as a target to dissect the mechanisms by which NETs activated keratinocytes. At the frontline of innate immune surveillance are the pattern recognition receptors (PRRs) which are known to function as receptors for some of the proteins enriched in NETs (33). Considering that NETs are web-like structures, we focused on PRRs on plasma membrane which were reported to be significantly increased in psoriatic lesions, including TLR2, Dectin-1, and TLR4 (33, 34). Our results showed that the expressions of TLR4 was up-regulated more than 4-fold in keratinocytes stimulated by NETs (Figure 4A). Moreover, TLR4 expression was increased and primarily located in the epidermis of psoriatic skin, but with weak epidermal expression in healthy controls (Figure 4B). The exposure of keratinocytes to NETs (5 μg/mL) markedly increased the expressions of MyD88, TNF receptor-associated factor 6 (TRAF6), and mitogen-activated protein kinase 1 (TAK1) that are critical for TLR4 to activate downstream signaling pathways expressions (Figure 4C; Figure S4A). Furthermore, several of NETs components can bind and activate TLR4 signaling as endogenous ligands including HMGB1, S100A8, S100A9, and HSP70 (35, 36), and these were all increased in psoriatic skin and prominently co-localized with neutrophil MPO (Figure S4B).

It has been reported that NF-κB regulates transcription of the LCN2 gene (37, 38). Therefore, we measured the phosphorylation level of NF-κB in keratinocytes co-cultured with NETs. Our data showed that phosphorylation of NF-κB triggered by NETs peaked at 30 min (Figure 4D; Figures S4C,D), and was substantially reduced in keratinocytes lacking TLR4 or MyD88 (Figure 4E; Figures S4E–G). Moreover, LCN2 expression in response to NETs was strongly inhibited in either TLR4-, MyD88-, or NF-κB-silenced keratinocytes (Figure 4F; Figures S4F–H).

We also determined if TLRs, such as TLR7 and TLR9 that can sense nuclear material of NETs, had a role in NETs induced keratinocyte activation. Using siRNA approaches, our results showed that the up-regulation of LCN2 and IL36G by NETs stimulation was independent of TLR7 or TLR9 signaling (Figure 4G; Figures S4I,J). These results confirm that TLR4/MyD88 is involved in NF-κB activation in response to NETs and leads to production of LCN2 in keratinocytes.

### TLR4 Activation Synergizes With IL-36γ to Induce Pro-inflammatory Gene Expressions

**Figure 5:**
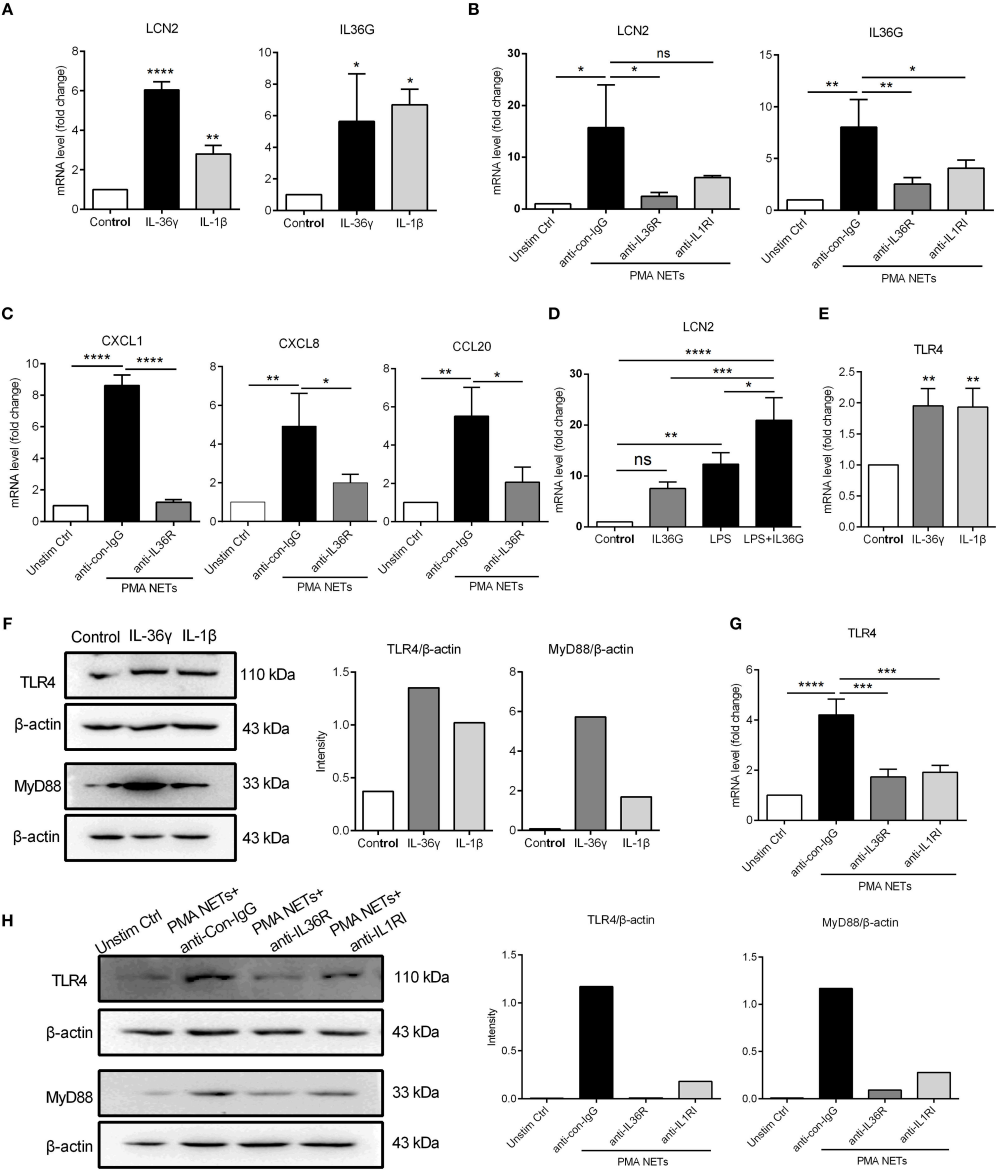
TLR4 activation synergizes with IL-36γ to induce inflammatory gene expression **(A)** The mRNA expressions of LCN2 and IL36G in keratinocytes stimulated with recombinant IL-36γ (100 ng/ml) or IL-1β (50 ng/ml) for 24 h. **(B,C)** The mRNA levels of LCN2, IL36γ, and other inflammatory mediators in keratinocytes co-cultured with NETs and anti-IL36R or not. **(D)** The mRNA expression of LCN2 in keratinocytes stimulated with IL-36γ and LPS. **(E,F)** The mRNA and protein levels of TLR4 and activation of MyD88 in cultured keratinocytes stimulated with IL-36γ or IL-1β for indicated time. **(G,H)** The mRNA and protein levels of TLR4 and activation of MyD88 in keratinocytes co-cultured with NETs and anti-IL36R or not for indicated time. *P < 0.05, **P < 0.01, ***P < 0.001, ****P < 0.0001, one-way ANOVA, *n* = 3 (mean ± SD). All the bars represent the average of three independent experiments.

Several reports have indicated an important role for IL-36, particularly IL-36γ, in psoriasis pathogenesis, as a potent pro-inflammatory mediator (39, 40), and as a valuable biomarker of disease activity (41). Recently, it is demonstrated that NETs associated proteases can process and activate IL-36 cytokines and amplify inflammatory responses in skin (28). Therefore, we assessed whether IL-36γ secreted by keratinocytes played a role in NETs-induced immune responses. QRT-PCR results showed that LCN2 and IL36G mRNA was highly induced in keratinocytes treated with bioactive IL-36γ or IL-1β (as a positive control) compared to unstimulated control (Figure 5A). Importantly, the up-regulation of LCN2, IL36G, CXCL1, CXCL8, and CCL20 in keratinocytes stimulated by NETs was largely inhibited by IL36R blockade (Figures 5B,C).

To determine a potential cross-talk between IL-36 and TLR4 signaling, we used low-dose LPS as a proxy for neutrophil-derived TLR4 endogenous ligands. QRT-PCR results demonstrated that IL-36γ synergized with TLR4 activation to promote increased LCN2 mRNA expression in keratinocytes (Figure 5D). In addition, both mRNA and protein expressions of TLR4, along with the adaptor molecule MyD88 were strongly induced by IL-36γ or IL-1β (as a positive control) (Figures 5E,F). Notably, IL36R blockade inhibited both TLR4 expression and MyD88 activation in keratinocytes co-cultured with NETs (Figures 5G,H). These data suggest that IL-36γ, secreted by NETs-stimulated keratinocytes, induces TLR4 expression, priming keratinocytes to respond to NETs, and subsequently synergizes with TLR4 activation to stimulate keratinocytes to highly express LCN2.

### Inhibition of TLR4 Function Ameliorates NETs-Exacerbated Skin Inflammation *in vivo*

**Figure 6:**
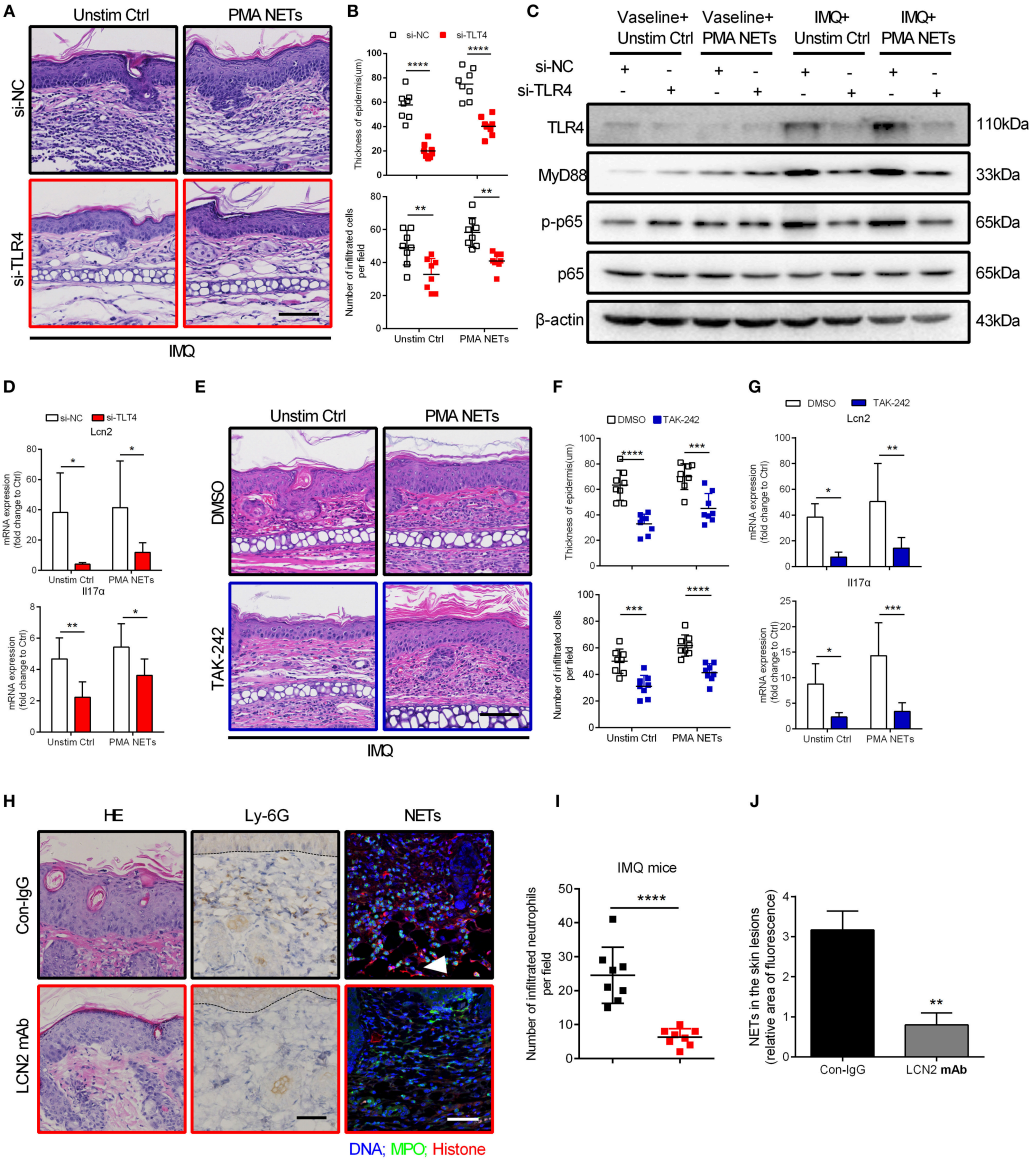
Inhibition of TLR4 function ameliorates NETs-exacerbated skin inflammation *in vivo* **(A)** Representative H&E analysis of the ears on day 7. TLR4 or control siRNA within the cream were applied to the ears of BALB/c mice daily, and NETs were subcutaneously injected back every 48 h. Scale bar = 100 μm. The study involved 3 mice per group and 3 independent experiments. **(B)** The analysis of skin thickening and inflammatory cell infiltration. Two-way ANOVA, *n* = 8 (mean ± SD). (**C)** Ear dermis and epidermis were separated. Western blot analysis of MyD88 and phosphorylated p65 expressions in the epidermis of ears after treatment as in A. **(D)** The mRNA level of Lcn2 and Il17α in the epidermis of mice subjected to indicated administration as in A. Two-way ANOVA. Values represent the means ± SD (*n* = 3). **(E)** Histological sections of mouse ears on day 7. TAK-242 or DMSO within the cream were applied to the inside ear of BALB/c mice daily, and NETs were subcutaneously injected back every 48 h. Scale bar = 100μm, *n* = 3 per group. (**F)** Statistical analysis of the epidermal thickness and infiltrated cells in dermis. Two-way ANOVA, *n* = 8 (mean ± SD). **(G)** Lcn2 and Il17α expressions in the epidermis of ears. Two-way ANOVA. Values represent the means ± SD (*n* = 3). **(H)** Representative H&E staining, infiltrated neutrophils labeled by Ly-6G, and immunofluorescence staining NETs structure in back skin from control IgG or LCN2 mAb treated IMQ mice, *n* = 5 per group. Scale bar: 100 μm. **(I)** Statistical analysis of Ly-6G labeled cells in H. Two-tailed Student's *t*-test, *n* = 8 (means ± SD). **(J)** Statistical analysis in back skins of control IgG or LCN2 mAb treated IMQ mice. Two-tailed Student's *t*-test, *n* = 3 (means ± SD). Scale bar = 100μm. *P < 0.05, **P < 0.01, ***P < 0.001, ****P < 0.0001. All the bars represent the average of three independent experiments.

Next, we explored the therapeutic potential of TLR4 inhibition for NETs-exacerbated psoriasis-like inflammation in the IMQ mouse model (Figure S5A). Topical treatment with siRNA targeting TLR4 for 7 consecutive days significantly attenuated acanthosis and immune cell infiltration in ears of the IMQ-treated group, with or without NETs injection (Figures 6A,B; Figures S5B,C). Likewise, expressions of downstream signaling molecules including MyD88 and phosphorylated NF-κB p65 were decreased accordingly (Figure 6C; Figure S5D). Consistent with these findings, qRT-PCR demonstrated decrease in mRNA expressions of Lcn2 and Il17A following TLR4 silencing (Figure 6D; Figure S5E). Furthermore, daily topical treatment with the TLR4 inhibitor TAK-242 also reduced the severe psoriasis phenotype in IMQ mouse model (Figures 6E–G). These *in vivo* results demonstrate that the TLR4/MyD88/NF-κB-dependent signaling pathway is aberrantly activated in keratinocytes by NETs, and that targeting of this pathway can lead to improvement in psoriasis-like inflammation.

To address the potential role of LCN2 in this process, we neutralized LCN2 using antibody in two mouse models of psoriasis (Figure S6A), the IMQ model and the transgenic K14-VEGF model (42, 43), and after 7 or 14 consecutive days, marked reduction in acanthosis and the number of Ly-6G labeled neutrophils was observed (Figures 6H,I; Figures S6B–D). Notably, administration of LCN2 neutralizing antibody significantly reduced formation of NETs in skin lesions of IMQ mice (Figures 6H,J). In summary, these data suggest that LCN2, secreted by activated keratinocytes and neutrophils, is a critical inflammatory factor down-stream of TLR4/IL-36R that participates in and sustains NETs-exacerbated psoriasis inflammation.

## Discussion

**Figure 7:**
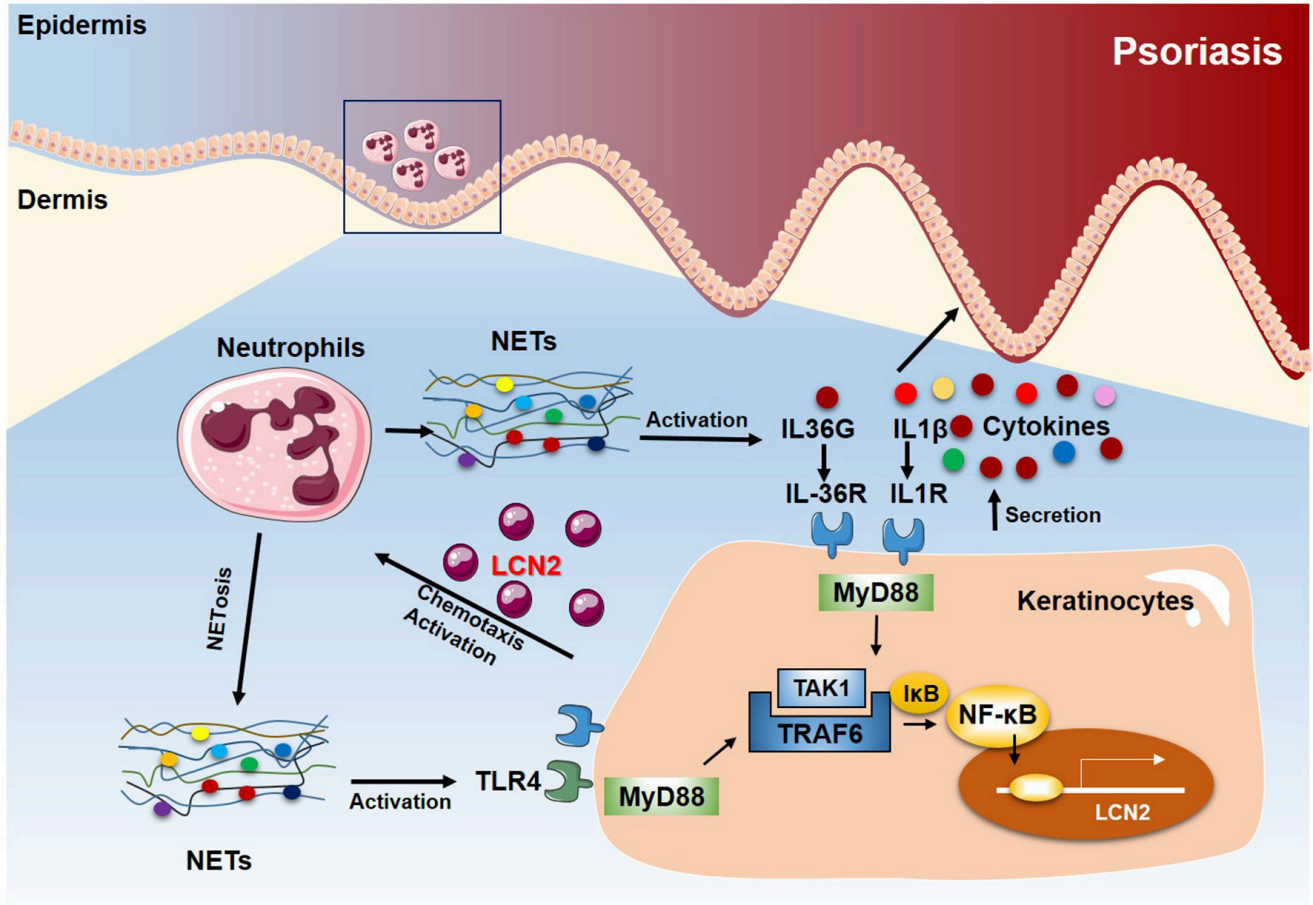
Proposed model of the NETs-TLR4/IL-36R-keratinocyte amplification loop in psoriasis. In psoriasis, neutrophils infiltrating the epidermis undergo NETosis in close proximity to keratinocytes. NETs stimulate keratinocytes to produce high levels of various inflammatory mediators, including LCN2, IL-36γ, CXCL8, and CXCL1. Secreted and activated IL-36γ induces TLR4 expression. Endogenous neutrophils-derived TLR4 ligands then synergize with IL-36, signaling through MyD88 and NF-kB activation, to induce LCN2 and IL-36γ production. In turn, the up-regulated LCN2 modulates NETs formation and neutrophil migration, enhancing and sustaining the inflammatory response. Thus, targeting NETs/TLR4 may provide a novel therapeutic approach for the treatment of psoriasis.

In this study, we confirm that circulating neutrophils are found in a pre-activated state in psoriasis, contributing to the high NETs level in patients. Furthermore, using a commonly used mouse model of psoriasis-like inflammation (44), we demonstrate that neutrophils have a major contribution to psoriasis-like inflammation heightening IL-17A and IL-36 responses, which is dependent upon NETs release and function. In addition, NETs interact directly with keratinocytes and activate an TLR4/IL-36R crosstalk with subsequent activation of the MyD88/NF-κB signaling pathway. This in turn leads to increased expressions of multiple pro-inflammatory cytokines and chemokines, most notably LCN2, which results in increased accumulation of immunocytes, including neutrophils, and amplification and sustenance of the inflammatory cascade in psoriasis (Figure 7).

The proteome of NETs, or “NETome,” has been reported to be regulated under different physiological conditions, which may affect their roles in inflammation (45). Many of the protein components of NETs, which have been explored in several studies (31, 46), including S100A8, S100A9, LCN2, and HSP70 that have been previously shown to have potent pro-inflammatory properties (17, 18, 47, 48). In contrast, our study shows minimal direct impact of these proteins on keratinocytes, and much less than that seen with intact NETs, suggesting that full integrity of NETs is required for eliciting their full pro-inflammatory effect in psoriasis. Several studies have addressed the effect of intact NETs on inflammatory responses. This includes activation of trypsin in acute pancreatitis (49, 50), priming of T cells through reduction in their activation threshold (51), and triggering of macrophages for cytokine release in atherosclerotic plaques (52). The studies that have looked at the role of NETs in psoriasis have been limited to plasmacytoid DCs (pDCs), demonstrating that NETs, through sensing of NETs derived DNA by TLR9, promote production of type I interferons to initiate inflammation in psoriasis (53, 54).

In psoriatic skin, NETs are mainly detected in close vicinity to keratinocytes, however, beyond one study showing induction of β-defensin-2 by NETs (23), no studies have addressed the broader effects of NETs on keratinocytes or the mechanisms involved. In striking contrast to pDCs, neither TLR7 or TLR9 contribute to NET induced activation of keratinocytes. In addition to being composed of DNA, histones, and S100A proteins, NETs carry a range of proteases, including cathepsin, elastase, and proteinase-3 (28). These proteases have been shown to activate IL-36 cytokines, which are expressed and secreted by keratinocytes as inactive precursor, thereby increasing their biologic activity about 500-fold (28). IL-36 cytokines are highly expressed in both chronic plaque psoriasis (55) and pustular psoriasis (39), and IL-36 signaling has been shown to be essential for development of psoriasis-like inflammation in the IMQ mouse model (56). This is consistent with our findings, which show that IL-36 signaling is necessary for the pro-inflammatory effect of NETs. Thus, NETs, through activation of IL-36 cytokines and IL-36R signaling, promote the expression of TLR4, which can bind endogenous ligands such as S100A8, S100A9, and HSP70 (57, 58) that are abundantly expressed in neutrophils and NETs. This IL-36/TLR4 cross-talk can explain the findings from a recent report that shows that targeting and inhibition of TLR4 prevents development of autoinflammatory symptoms in a mouse model of pustular psoriasis caused by deficiency of the IL-36 receptor antagonist (59), but mutations in the IL-36 receptor antagonist have been shown to predispose to generalized pustular psoriasis (60, 61).

LCN2 is a prominently expressed gene in psoriasis (19, 62), and is one of the genes most prominently induced by NETs in keratinocytes. In our previous work, we have demonstrated that LCN2 potentiates inflammation by acting as a chemoattractant and as a trigger for neutrophil activation in psoriasis (19). Importantly, IMQ mice treated with neutralizing LCN2 antibody had decreased number of neutrophils, as well as decreased number of NETs in inflamed skin. Therefore, neutrophils, through release of NETs in the epidermis, set up a self-sustaining loop in which increased expression and secretion of LCN2, by NETs-stimulated keratinocytes, continuously attracts more neutrophils into the skin, leading to even greater increase in NETs levels in skin, thereby propagating the inflammatory process. This is likely to both amplify and sustain neutrophil-driven inflammatory responses in psoriasis and pustular psoriasis.

In addition to providing novel insights into neutrophils immune responses in skin, our findings may also offer novel therapeutic targets for psoriasis. Dimethyl fumarate, which has been used to treat psoriasis successfully, showed inhibitory effect on NETs formation (63). Target PAD4 to inhibit NETs formation has recently been shown to be protective in murine model of lupus, diabetes, and atherosclerosis, without any notable adverse events (30, 64, 65). In addition, DNase 1 treatment, that breaks up NETs structures may also have a therapeutic potential (66, 67). In our mouse experiments, both CI-amidine and DNase I treatment improved psoriasis-like manifestations, confirming that NETs can be a treatment target for psoriasis patients. In addition, as seen with the marked improvement in our model with both TLR4 inhibition, and LCN2 blockade, these provide additional novel therapeutic targets in both chronic plaque and pustular psoriasis.

In conclusion, we demonstrate NETs as the critical link between neutrophil infiltration and keratinocyte activation in psoriasis. As neutrophil infiltration into epidermis is found in many other inflammatory conditions, including lupus and bullous pemphigoid, the findings described here are likely to have implications beyond psoriasis alone. Furthermore, our data provide a rational basis for development of therapies specifically targeting the autoinflammatory loop of NETs-keratinocytes-IL-36 in psoriasis and identify TLR4 as novel therapeutic targets in psoriasis.

## Ethics Statement

All mouse experiments described in this study were approved by the institutional review board and carried out in accordance with the National Institutes of Health guide for the care and use of Laboratory animals. The protocol was approved by the committee of the Fourth Military Medical University. All efforts were made to minimize suffering and ensure the highest ethical and humane standards. All analyses of human materials were performed in full agreement with institutional guidelines, with the approval of the Ethical committee of the Fourth Military Medical University (reference number KY20173053-1) and conducted according to the principles in the Declaration of Helsinki. Informed consent to collect blood and skin lesions were obtained from all subjects enrolled in the study.

## Author Contributions

ShuS and ED designed the experiments, analyzed the data, and wrote the manuscript. ShuS and HF performed most of the experiments and analyzed the data. BL and KX participated in the cell experiments. HQ helped with the statistical analyses of some data. JZ, YZ, SheS and TC helped some of the mouse experiments. TZ and PQ performed ELISA detecting inflammatory mediators. CL provided patient samples and data. JG and GW conceived and supervised this study, provided critical suggestions and discussions throughout the study, and revised the manuscript.

### Conflict of Interest Statement

The authors declare that the research was conducted in the absence of any commercial or financial relationships that could be construed as a potential conflict of interest.
