# Supplementary figures and images for: Neutrophil Extracellular Traps Promote Inflammatory Responses in Psoriasis via Activating Epidermal TLR4/IL-36R Crosstalk
Source: Front Immunol. 2019 Apr 5;10:746. doi: 10.3389/fimmu.2019.00746 (PMC6460719; doi:10.3389/fimmu.2019.00746)

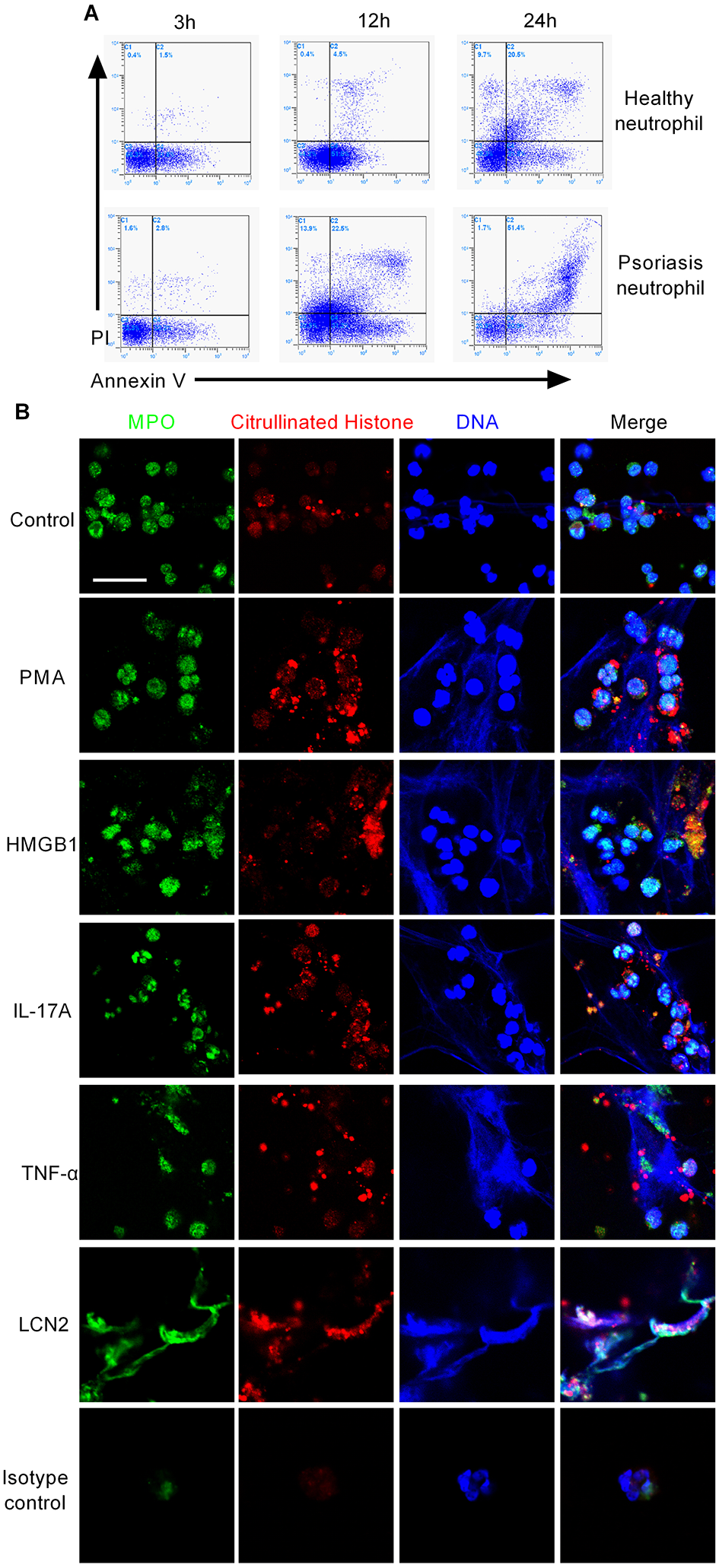

Supplement: Supplementary file 1 [file Image_1.TIF]

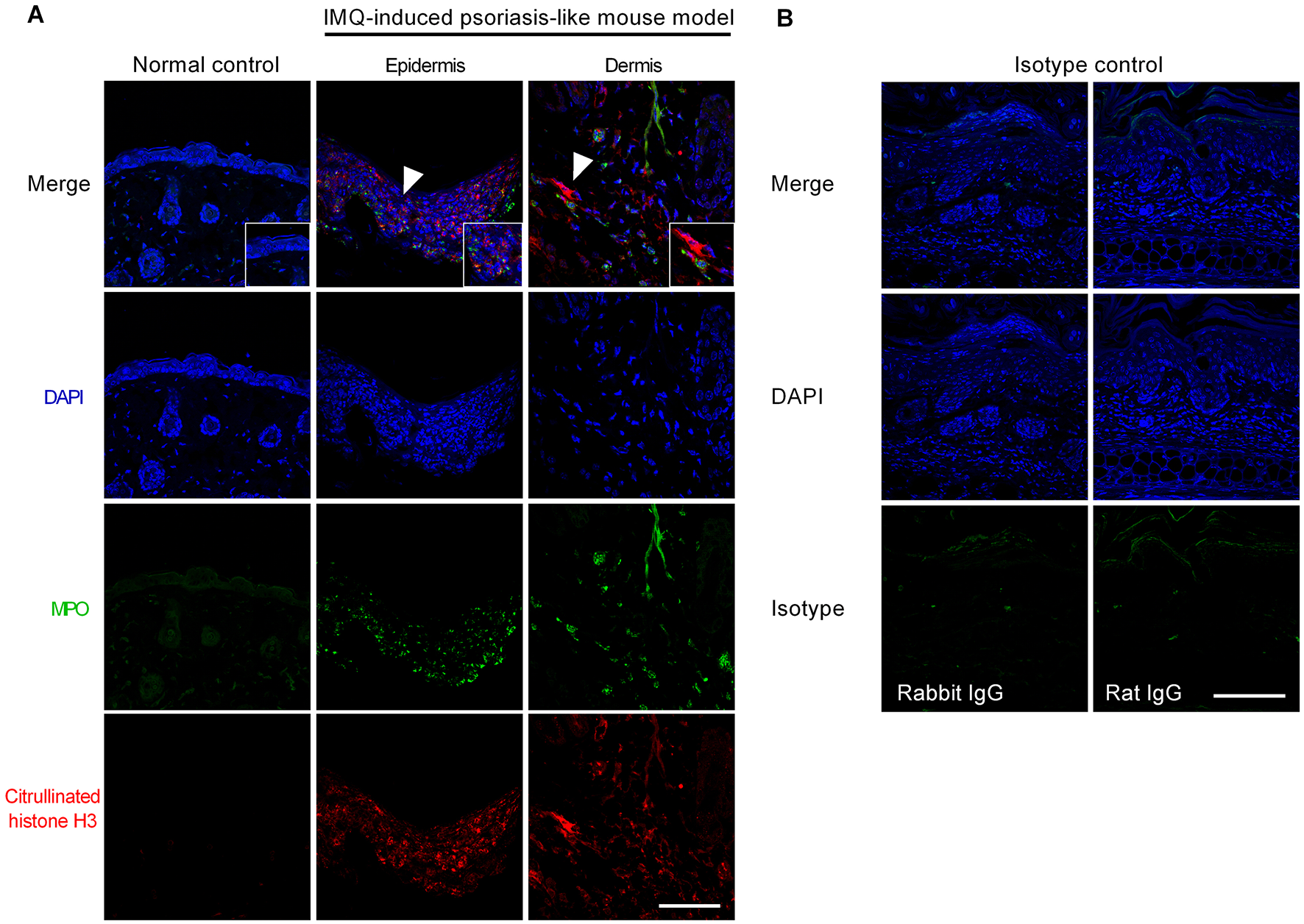

Supplement: Supplementary file 2 [file Image_2.TIF]

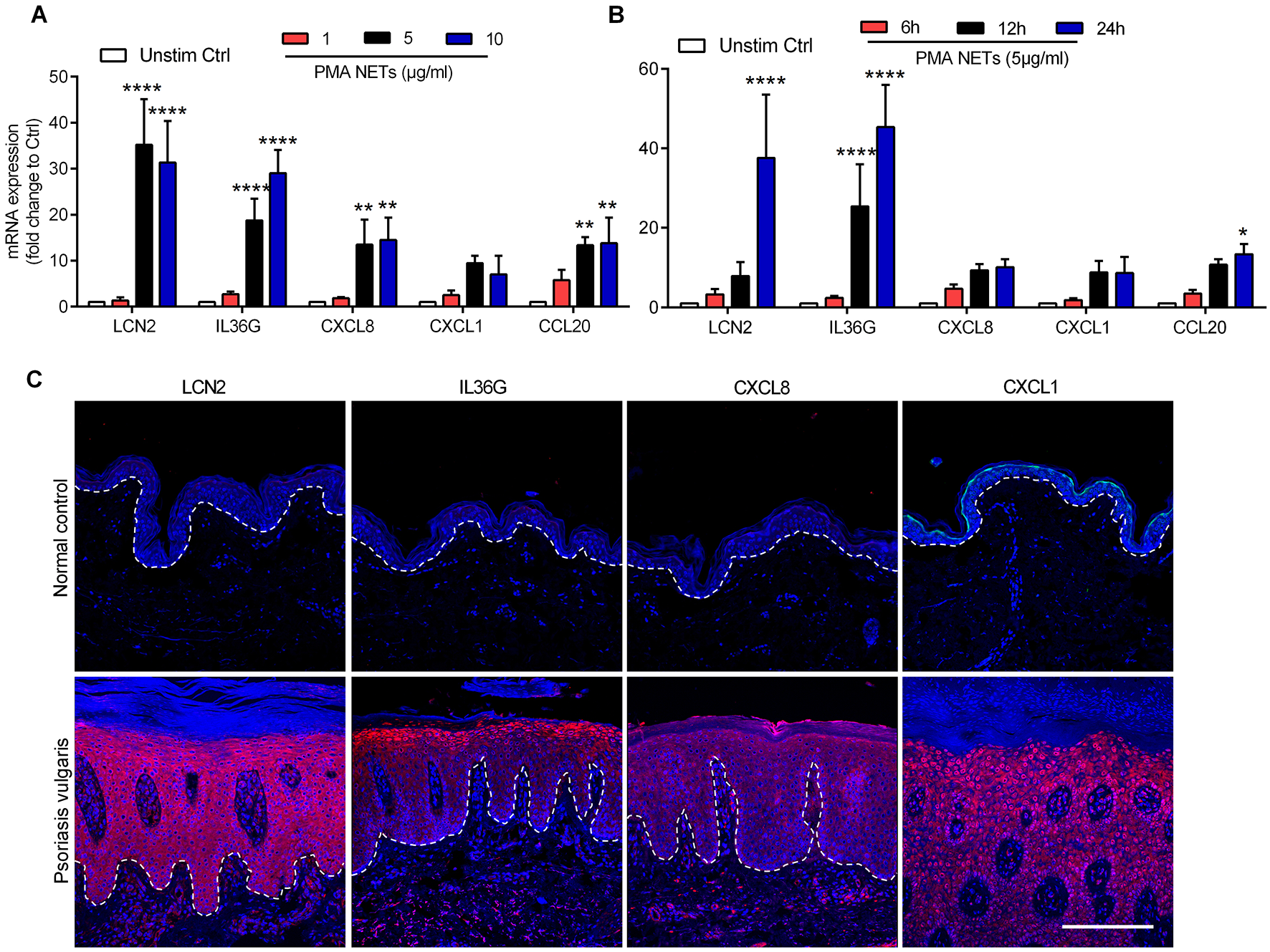

Supplement: Supplementary file 3 [file Image_3.TIF]

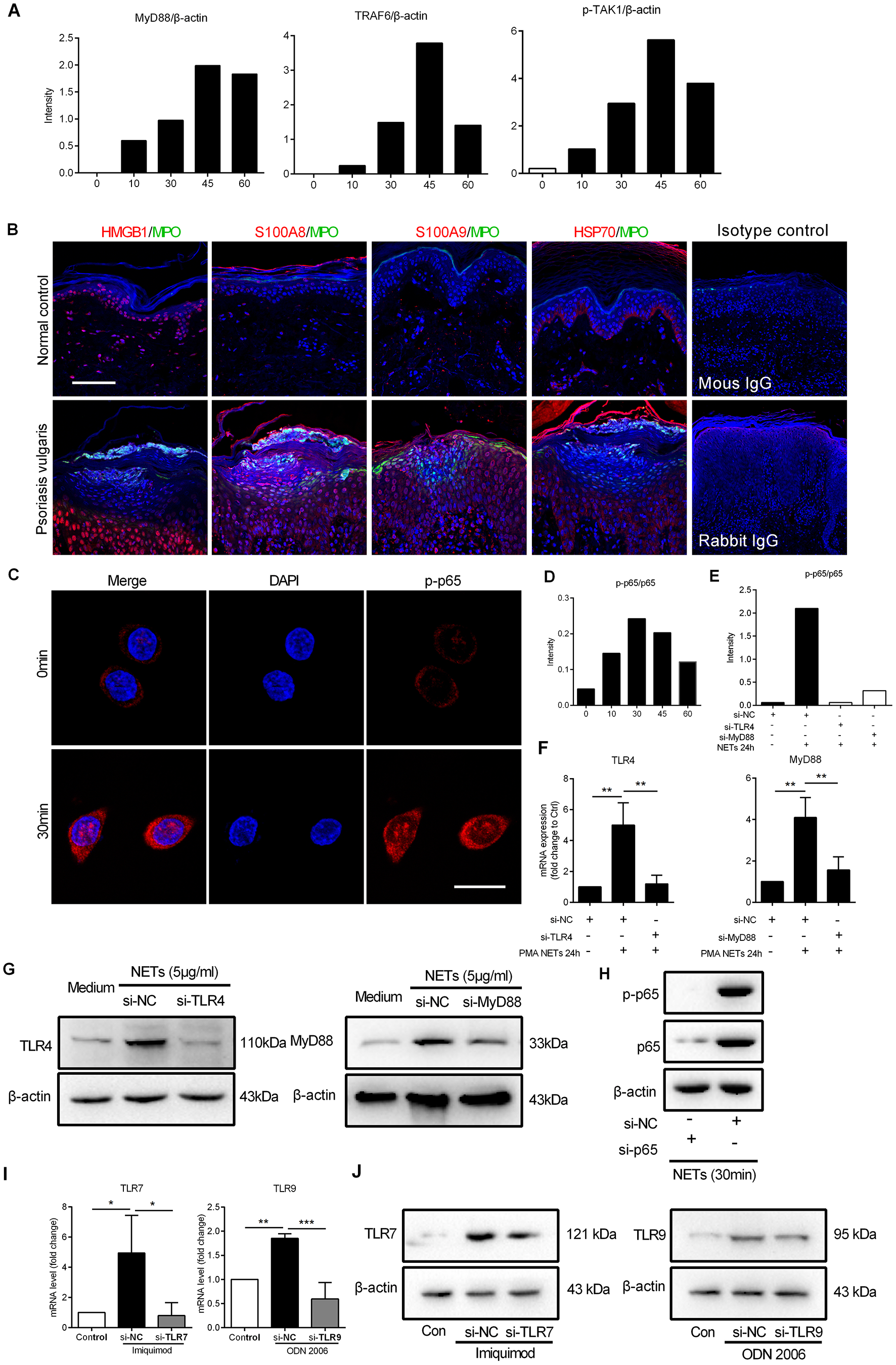

Supplement: Supplementary file 4 [file Image_4.TIF]

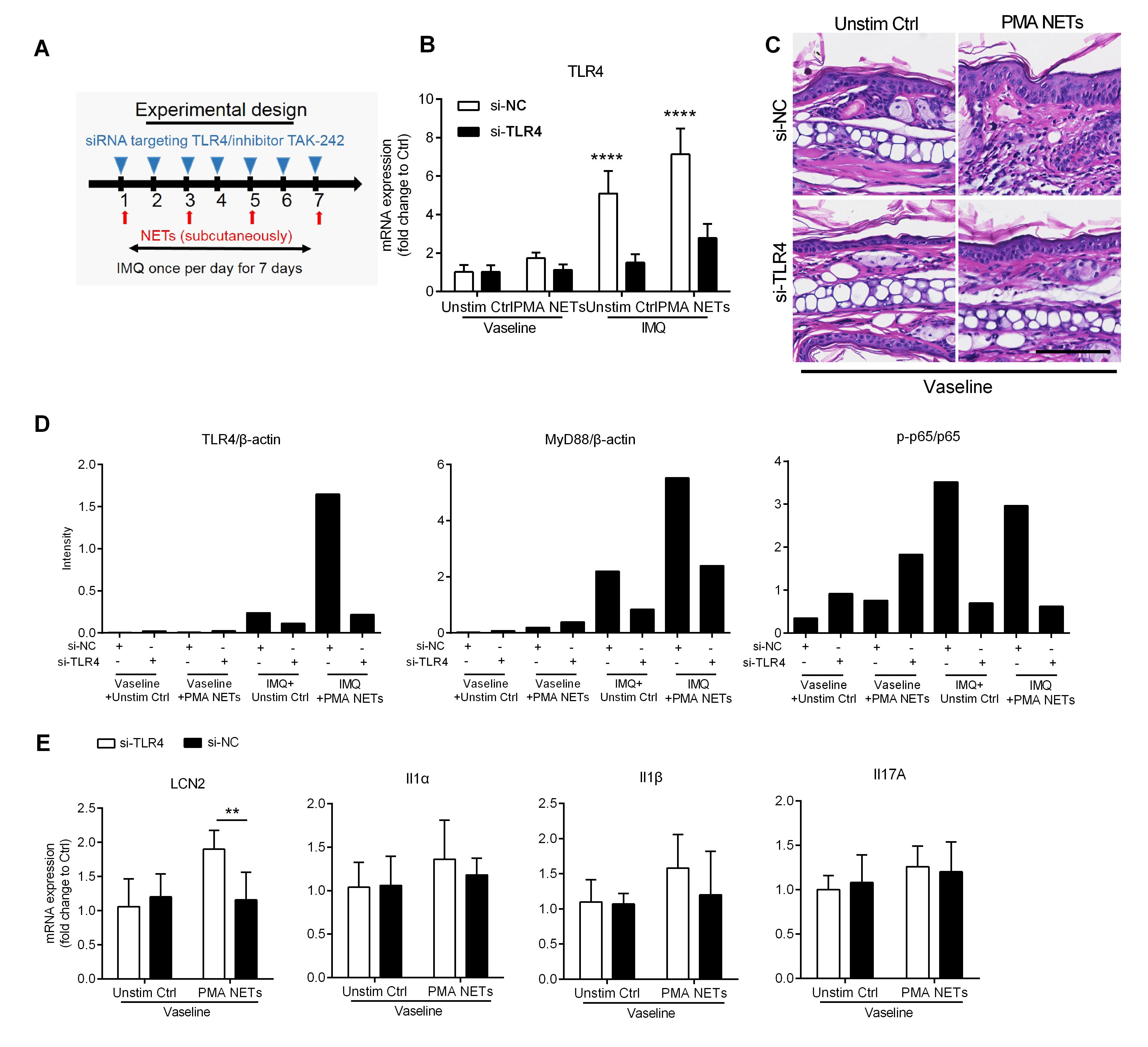

Supplement: Supplementary file 5 [file Image_5.TIF]

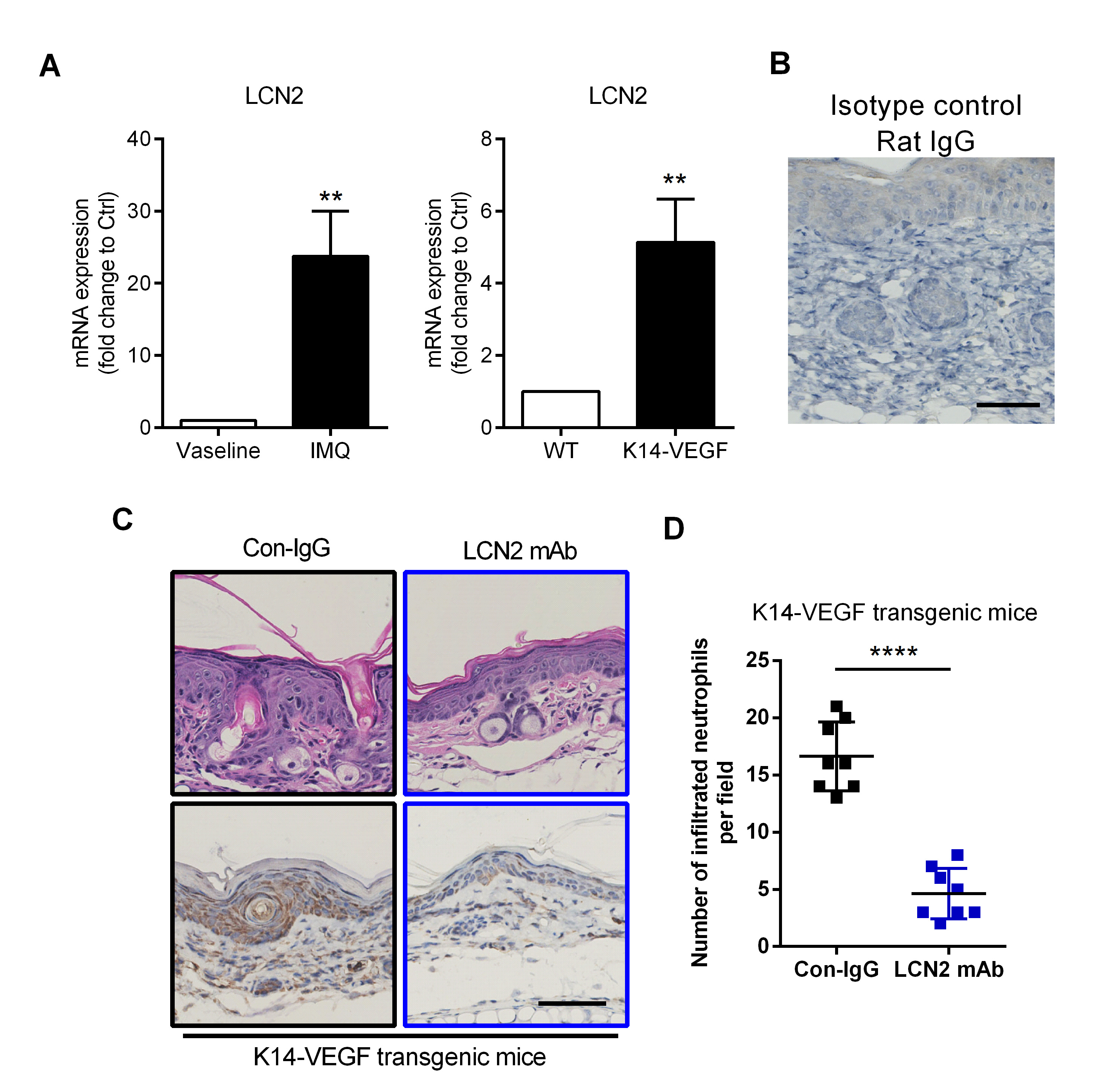

Supplement: Supplementary file 6 [file Image_6.TIF]
