# Supplementary material for: Neutrophil Extracellular Traps Promote Inflammatory Responses in Psoriasis via Activating Epidermal TLR4/IL-36R Crosstalk
Source: Front Immunol. 2019 Apr 5;10:746. doi: 10.3389/fimmu.2019.00746 (PMC6460719; doi:10.3389/fimmu.2019.00746)
Supplement: Supplementary file 7 [file Data_Sheet_1.pdf]

Supplemental Materials for

Neutrophil Extracellular Traps Promote Inflammatory Responses in Psoriasis via activating Epidermal TLR4/IL-36R Crosstalk

Supplemental methods

Neutrophil isolation

The separation of peripheral neutrophils was performed using the method of density gradient separation as described previously (1).

RNA isolation, cDNA synthesis, and quantitative real-time polymerase chain reaction (qRT-PCR)

The mRNA expressions of cytokines, chemokines, other inflammatory mediators, receptors, and β-actin were quantified in triplicate by SYBR Green qRT-PCR. QRT-PCR was performed using on a CFX384 Real-Time PCR Detection System (Biorad Laboratories). β-Actin was used as the endogenous control in the RNA analysis of mouse skin and human keratinocytes. All values of neutrophils in Figure 2 were normalized to the expression of the housekeeping gene SDHA. The primers are listed in Supplemental Table 2. Relative quantification was performed according to the ΔΔCT method, and results were expressed in the linear form using the formula 2-ΔΔCT.

Tissue immunofluorescence and immunohistochemical staining

For immunofluorescence staining, skin sections were fixed, stained, and imaged using confocal microscopy. To evaluate the location of NETs, mouse skin tissues were incubated with the specific primary antibodies for MPO (1:150, Abcam), citrullinated-

histone H3 (1:150, Abcam), and Hoechst 33342 (1:1000, Sigma). Tissue slides were incubated with anti-CD4 (1:100, Abcam), anti-Ly-6G (1:500, Biolegend), anti-TLR4 (1:100, Santa Cruz Biotechnology), anti-LCN2 (1:100, Abcam), anti-IL-36γ (1:50, Proteintech), anti-CXCL8 (1:100, R&D), anti-CXCL1 (1:300, Abcam), anti-HMGB1 (1:100, Abcam), anti-S100A8 (1:100, Sino Biological), anti-S100A9 (1:100, Proteintech), and anti-HSP70 (1:100, Abcam) to determine the expression and location of these proteins. Cy3-conjugated goat anti-rabbit, anti-rat, and anti-mouse IgG antibodies (1:500; Abcam) and FITC-conjugated goat anti-rabbit and anti-mouse IgG antibody (1:200, CWBIO) were used as secondary antibodies. Nuclear DNA was detected by incubating cells with Hoechst 33342 (1:1000, Sigma) for 20 min at room temperature. Confocal images were acquired using Olympus Fluoview 1000 microscope with a PlanApo N (×40 with and without a 2.5 digital zoom).

For immunohistochemistry of mouse samples, 4μm sections of paraffin embedded skins were blocked at room temperature with 5% goat serum in PBS for 30 min, and incubated with rat anti-Ly-6G (1:500, Biolegend) at 4°C overnight, followed by HRP labeled goat anti-rat antibodies (1:100, CWBIO) for 30 min at room temperature. DAB revelation (Gene tech) was used for detecting the biotinylated antibodies.

H&E staining and analysis

Normal and lesional skin from donors and mice were fixed in 4% paraformaldehyde, embedded in paraffin, sliced, and stained with hematoxylin and eosin for histological analysis. Epidermal thickness represents the maximal epidermal thickness as measured from the junction of the stratum corneum to the deepest portion

of the rete ridge (in humans) or inter-follicular dermis (in mice). Number of the infiltrated inflammatory cells in dermis was counted in eight fields (40μm×40μm) per section, which was manually counted by two independent blinded observers.

NETs immunofluorescent staining

Neutrophils were seeded in poly-L-lysine coated coverslips and incubated at 37˚C, 5% CO_2 before stimulation with PMA (50nM, Sigma), recombinant HMGB1 (500ng/ml, Sigma), LCN2 (1μg/ml, Abcam), TNF-α (50ng/ml, Peprotech), or IL-17A (50ng/ml, Peprotech) for 4 hours at 37˚C. Cells were washed with ice-cold PBS, and fixed immediately with 4% paraformaldehyde for 15 min, and then permeabilized with 0.1% Triton for 20 minutes. Cells were then incubated with MPO (1:100, Abcam) and citrullinated-histone H3 (1:100, Abcam) overnight at 4˚C, followed by incubation with secondary fluorochrome-conjugated antibodies (Abcam) for 2h at room temperature. Nuclear DNA was detected by incubating cells with Hoechst 33342 (1:1000, Sigma) for 20 min at room temperature. Confocal images were acquired using Olympus Fluoview 1000 microscope with a PlanApo N (×40 with and without a 2.5 digital zoom).

Flow cytometry

To evaluate the neutrophil spontaneous death, peripheral neutrophils were collected and isolated from psoriasis patients and healthy controls by Miltenyi column. Human neutrophils were then cultured for the 3h, 12h, and 24h. Cells were then harvested, washed twice with ice-cold PBS, and stained with human annexin V (FITC labeled) first and then PI following a protocol provided by the manufacturer (Annexin V-FITC/PI Detection Kit; 7 Seabiotech). FACS was performed using a FACS Canto

flow cytometer (BD). In this FACS analysis, cell debris were eliminated by appropriate gating on forward and side scatter. Neutrophil spontaneous death was calculated as the percentage of PI+ cells at each time point.

Small interfering RNA

The small interfering RNA (siRNA) for human TLR4, TLR7, TLR9, MyD88, NF-κB, and control siRNA were synthesized from Ribobio. Keratinocytes were processed with siRNA (5nM) and Lipofectamine 3000 (Invitrogen) following the instructions inside the siRNA kit. After 48h for gene silencing, NETs at indicated concentration were added to each well for stimulation.

Western blot analysis

Western blot assays were performed using whole cell lysates from primary keratinocytes. In short, lysates prepared from cells in a lysis buffer containing a protease inhibitor cocktail (Roche) and a phosphatase inhibitor cocktail (Thermo Fisher Scientific) were separated with SDS-polyacrylamide gel electrophoresis gels. The following antibodies were used: Membranes were incubated overnight using the following antibodies: NF-κB and phospho-NF-κB (1:1000, Cell Signaling Technology), TLR4 (1:100, Santa Cruz Biotechnology), MyD88 (1:1000, Cell Signaling Technology), TRAF6 (1:800, Proteintech), p-TAK1 (1:1000, Cell Signaling Technology), LCN2 (1:1000, Abcam), and β-actin (1:5000, CWBIO) as an internal control.

ELISA

LCN2, CXCL1, CXCL8, and IL-36γ levels in the cell-free supernatants from non-stimulated or NETs-stimulated keratinocytes were measured using ELISA assay kits

(Neobioscience) following the instructions inside the ELISA kits. Supernatants were stored at -20°C until use for ELISA.

Trypan blue

To determine cell viability of peripheral neutrophils isolated from psoriasis patients and healthy controls, we performed trypan blue exclusion assays according to the instructions (Thermo Fisher Scientific). Experiments were repeated for three times.

Reference

1. Gonzalez AS, Bardoel BW, Harbort CJ, Zychlinsky A. Induction and quantification of neutrophil extracellular traps. *Methods Mol Biol* (2014) 1124(3)07-18. doi:10.1007/978-1-62703-845-4_20

Supplemental figure legends

Supplemental Figure 1 (related to Figure 1) Mature psoriasis neutrophils undergo accelerated death and form NETs

(**A**) Representative flow cytometry analysis of Annexin V-FITC/PI in peripheral neutrophils of psoriasis patients and healthy controls. Neutrophils were isolated and cultured in RPMI-1640 growth medium supplemented with 12% fetal bovine serum for indicated time. (**B**) Representative immunofluorescence image of NETs structures stained by extranuclear DNA (blue), extracellular MPO (green), and citrullinated histone (red). NETs released from psoriasis neutrophils were triggered by PMA (50nM), IL-17A (50ng/ml), TNF-α (50ng/ml), HMGB1 (500ng/ml), and LCN2 (1μg/ml) for 4 hours. Scale bars, 50μm.

Supplemental Figure 2 (related to Figure 2) The NETs structure is observed in skin lesions of IMQ-induced mouse model

(A) Enhanced NETosis (white solid arrows) in the epidermis and dermis of IMQ-induced mouse model was co-stained by extranuclear DNA (blue), extracellular MPO (green), and citrullinated histone (red). (**B**) Representative immunofluorescence image of isotype control for Figure 2D. Scale bar, 100μm.

Supplemental Figure 3 (related to Figure 3) NETs activate inflammatory responses in keratinocytes *in vitro*

(**A, B**) QRT-PCR for LCN2, IL36G, CXCL8, CXCL1, and CCL20 with cDNA from cultured primary human keratinocytes stimulated with NETs at different concentration for indicated time. Data are expressed as means ± SD (n=3). Two-way ANOVA. *P <

0.05, **P < 0.01, ****P < 0.0001. (**C**) Immunofluorescent staining of healthy skin and psoriatic lesions for LCN2, IL-36γ, CXCL1, and CXCL8. Nuclei were stained with Hoechst 33342. Scale bars, 100μm.

Supplemental Figure 4 (related to Figure 4) TLR4/MyD88/TRAF6/TAK1 and NF-κB signaling mediate the NETs-induced immune responses

(**A**) Quantitative analysis of western blot result in Figure 4C. (**B**) Immunofluorescent co-staining of MPO and endogenous TLR4 ligands in healthy skin and psoriatic lesions. Nuclei were stained with Hoechst 33342. Scale bars, 100μm. (**C**) The expression of p-p65 in NETs-stimulated keratinocytes was monitored by immunofluorescence. Bar, 10μm. (**D, E**) Quantitative analysis of western blot result in Figure 4D, E. (**F, G**) The efficiency of TLR4 or MyD88 silencing in keratinocytes. One-way ANOVA. n=3 (mean±SD). (**H**) The successful silence of p65 in keratinocytes. (**I, J**) The efficiency of TLR7 or TLR9 silencing in keratinocytes. One-way ANOVA. n=3 (mean±SD). *P < 0.05, **P < 0.01, ***P < 0.001. All the bars represent the average of three independent experiments.

Supplemental Figure 5 (related to Figure 6) TLR4 silencing by siRNA in IMQ-induced mice

(**A**) Scheme of experimental procedure for siRNA or TAK-242 targeting TLR4 silencing in the epidermis of IMQ treated mice with or without subcutaneous NETs injection. (**B**) The effective silencing of TLR4 in the ears of IMQ treated mice. ****P < 0.0001, two-tailed Student's t-test, n = 3 per group (mean ± SD). (**C**) Representative H&E staining of control mice treated with control or TLR4 siRNA

with subcutaneous injection of NETs. These control mice were applied with Vaseline every day. Scale bar, 100µm. (**D**) Quantitative analysis of western blot result in Figure 6C. (**E**) The mRNA expressions of LCN2 and inflammatory cytokines in the ears of Vaseline-treated mice treated as in C. Data are presented as the mean ± SD (n=3), two-tailed Student's t-test, **$P < 0.01$. All the bars represent the average of three independent experiments.

Supplemental Figure 6 (related to Figure 6) Neutralizing LCN2 with antibody in K14-VEGF mice alleviates the psoriasis-like inflammation

(**A**) The expression of LCN2 in the skin samples from IMQ mice (left panel) and K14-VEGF mice (right panel). Two-tailed Student's t-test. Data are expressed as mean ± SD (n=3). **$P < 0.01$. (**B**) Representative IHC image of isotype control for Figure 6H (**C, D**) Representative H&E staining and infiltrated neutrophils labeled by Ly-6G in back skin from control IgG or LCN2 mAb treated K14-VEGF mice, n = 5 per group. Scale bar: 100 µm. Two-tailed Student's t-test, n = 8 (means ± SD). ****$P < 0.0001$.

Supplemental table1. List of identified NETs proteins.

Gene ID	Gene Symbol	Description	Coverage	MW [kDa]
6280	S100A9	protein S100-A9 [Homo sapiens]	70.18	13.2
4627	MYH9	myosin-9 [Homo sapiens]	31.22	226.4
87	ACTN1	alpha-actinin-1 isoform b [Homo sapiens]	34.42	103.0
2316	FLNA	PREDICTED: filamin-A isoform X2 [Homo sapiens]	17.80	276.4
81	ACTN4	alpha-actinin-4 isoform 1 [Homo sapiens]	33.48	104.8
3936	LCP1	plastin-2 [Homo sapiens]	37.64	70.2
4057	LTF	lactotransferrin isoform 2 [Homo sapiens]	32.28	73.1
7086	TKT	transketolase isoform 1 [Homo sapiens]	28.09	67.8
309	ANXA6	annexin A6 isoform 2 [Homo sapiens]	36.66	72.4
309	ANXA6	PREDICTED: annexin A6 isoform X1 [Homo sapiens]	34.33	75.2
847	CAT	catalase [Homo sapiens]	35.86	59.7
301	ANXA1	annexin A1 [Homo sapiens]	42.77	38.7
7414	VCL	vinculin isoform VCL [Homo sapiens]	24.95	116.6
3043	HBB	hemoglobin subunit beta [Homo sapiens]	68.71	16.0
4353	MPO	myeloperoxidase precursor [Homo sapiens]	20.00	83.8
7094	TLN1	talin-1 [Homo sapiens]	10.67	269.6
3303	HSPA1A	heat shock 70 kDa protein 1A [Homo sapiens]	23.56	70.0
306	ANXA3	annexin A3 [Homo sapiens]	47.06	36.4
6709	SPTAN1	spectrin alpha chain, non-erythrocytic 1 isoform 3 [Homo sapiens]	9.91	282.1
2821	GPI	glucose-6-phosphate isomerase isoform 4 [Homo sapiens]	25.85	60.1
3040	HBA2	hemoglobin subunit alpha [Homo sapiens]	61.97	15.2
226	ALDOA	fructose-bisphosphate aldolase A isoform 1 [Homo sapiens]	33.52	39.4
71	ACTG1	actin, cytoplasmic 2 [Homo sapiens]	32.80	41.8
5230	PGK1	phosphoglycerate kinase 1 [Homo sapiens]	37.41	44.6
6279	S100A8	protein S100-A8 isoform d [Homo sapiens]	48.39	10.8

7431	VIM	vimentin [Homo sapiens]	22.75	53.6
3045	HBD	hemoglobin subunit delta [Homo sapiens]	54.42	16.0
3312	HSPA8	heat shock cognate 71 kDa protein isoform 1 [Homo sapiens]	24.61	70.9
10487	CAP1	PREDICTED: adenylyl cyclase-associated protein 1 isoform X2 [Homo sapiens]	15.40	51.8
5836	PYGL	glycogen phosphorylase, liver form isoform 2 [Homo sapiens]	22.88	93.1
3320	HSP90AA1	heat shock protein HSP 90-alpha isoform 2 [Homo sapiens]	22.68	84.6
3939	LDHA	L-lactate dehydrogenase A chain isoform 1 [Homo sapiens]	26.20	36.7
10109	ARPC2	actin-related protein 2/3 complex subunit 2 [Homo sapiens]	32.00	34.3
5216	PFN1	profilin-1 [Homo sapiens]	62.86	15.0
5223	PGAM1	phosphoglycerate mutase 1 isoform 1 [Homo sapiens]	40.55	28.8
6888	TALDO1	transaldolase [Homo sapiens]	24.63	37.5
213	ALB	serum albumin preproprotein [Homo sapiens]	14.29	69.3
3326	HSP90AB1	heat shock protein HSP 90-beta isoform c [Homo sapiens]	20.31	82.3
1992	SERPINB1	PREDICTED: leukocyte elastase inhibitor isoform X2 [Homo sapiens]	30.61	37.6
7167	TPI1	triosephosphate isomerase isoform 1 [Homo sapiens]	32.13	26.7
4478	MSN	PREDICTED: moesin isoform X3 [Homo sapiens]	16.78	66.6
10096	ACTR3	actin-related protein 3 isoform 2 [Homo sapiens]	29.70	42.0
3934	LCN2	neutrophil gelatinase-associated lipocalin precursor [Homo sapiens]	35.35	22.6
397	ARHGDIB	rho GDP-dissociation inhibitor 2 [Homo sapiens]	26.87	23.0
2539	G6PD	glucose-6-phosphate 1-dehydrogenase isoform b [Homo sapiens]	18.06	59.2
58	ACTA1	actin, alpha skeletal muscle [Homo sapiens]	20.16	42.0
6036	RNASE2	non-secretory ribonuclease precursor [Homo sapiens]	9.32	18.3
4048	LTA4H	leukotriene A-4 hydrolase isoform 2	21.64	66.8

		[Homo sapiens]		
3892	KRT86	keratin, type II cuticular Hb6 [Homo sapiens]	10.70	53.5
554313	HIST2H4B	histone H4 [Homo sapiens]	50.49	11.4
2934	GSN	gelsolin isoform b [Homo sapiens]	15.05	80.6
8826	IQGAP1	ras GTPase-activating-like protein IQGAP1 [Homo sapiens]	7.72	189.1
2665	GDI2	PREDICTED: rab GDP dissociation inhibitor beta isoform X1 [Homo sapiens]	29.55	48.3
5004	ORM1	alpha-1-acid glycoprotein 1 precursor [Homo sapiens]	33.83	23.5
9948	WDR1	WD repeat-containing protein 1 isoform 1 [Homo sapiens]	18.15	66.2
302	ANXA2	annexin A2 isoform 2 [Homo sapiens]	30.38	38.6
5657	PRTN3	myeloblastin precursor [Homo sapiens]	20.70	27.8
4318	MMP9	matrix metalloproteinase-9 preproprotein [Homo sapiens]	14.57	78.4
4860	PNP	purine nucleoside phosphorylase [Homo sapiens]	34.60	32.1
4190	MDH1	malate dehydrogenase, cytoplasmic isoform MDH1 [Homo sapiens]	20.96	36.4
4001	LMNB1	lamin-B1 isoform 1 [Homo sapiens]	15.19	66.4
25801	GCA	PREDICTED: grancalcin isoform X7 [Homo sapiens]	36.36	22.1
7534	YWHAZ	14-3-3 protein zeta/delta [Homo sapiens]	30.20	27.7
10097	ACTR2	actin-related protein 2 isoform b [Homo sapiens]	19.04	44.7
4046	LSP1	lymphocyte-specific protein 1 isoform 1 [Homo sapiens]	18.29	37.2
6711	SPTBN1	PREDICTED: spectrin beta chain, non-erythrocytic 1 isoform X2 [Homo sapiens]	4.98	272.7
4830	NME1	nucleoside diphosphate kinase A isoform b [Homo sapiens]	46.71	17.1
5226	PGD	6-phosphogluconate dehydrogenase, decarboxylating isoform 3 [Homo sapiens]	17.66	51.8
10135	NAMPT	nicotinamide phosphoribosyltransferase precursor [Homo sapiens]	15.48	55.5
3945	LDHB	L-lactate dehydrogenase B chain	28.74	36.6

		isoform LDHB [Homo sapiens]		
7791	ZYX	PREDICTED: zyxin isoform X2 [Homo sapiens]	12.38	57.7
1116	CHI3L1	chitinase-3-like protein 1 precursor [Homo sapiens]	24.02	42.6
832	CAPZB	F-actin-capping protein subunit beta isoform 1 [Homo sapiens]	22.06	30.6
2023	ENO1	alpha-enolase isoform 1 [Homo sapiens]	19.82	47.1
230	ALDOC	fructose-bisphosphate aldolase C [Homo sapiens]	14.29	39.4
2950	GSTP1	glutathione S-transferase P [Homo sapiens]	32.86	23.3
4317	MMP8	neutrophil collagenase isoform 1 preproprotein [Homo sapiens]	17.77	53.4
5478	PPIA	peptidyl-prolyl cis-trans isomerase A isoform 1 [Homo sapiens]	21.82	18.0
9555	H2AFY	core histone macro-H2A.1 isoform 2 [Homo sapiens]	16.17	39.5
25796	PGLS	6-phosphogluconolactonase [Homo sapiens]	34.88	27.5
1476	CSTB	cystatin-B [Homo sapiens]	45.92	11.1
654364	NME1-NME2	NME1-NME2 protein [Homo sapiens]	38.95	30.1
7170	TPM3	tropomyosin alpha-3 chain isoform 8 [Homo sapiens]	19.38	26.4
11151	CORO1A	coronin-1A [Homo sapiens]	11.71	51.0
3684	ITGAM	PREDICTED: integrin alpha-M isoform X1 [Homo sapiens]	7.15	120.4
4637	MYL6	myosin light polypeptide 6 isoform 1 [Homo sapiens]	22.52	16.9
9230	RAB11B	ras-related protein Rab-11B [Homo sapiens]	34.86	24.5
7408	VASP	PREDICTED: vasodilator-stimulated phosphoprotein isoform X3 [Homo sapiens]	9.79	39.5
3240	HP	haptoglobin isoform 1 preproprotein [Homo sapiens]	17.49	45.2
10562	OLFM4	olfactomedin-4 precursor [Homo sapiens]	14.90	57.2
8407	TAGLN2	transgelin-2 isoform b [Homo sapiens]	29.65	22.4
3068	HDGF	hepatoma-derived growth factor isoform e [Homo sapiens]	20.67	22.9
2664	GDI1	rab GDP dissociation inhibitor alpha	18.34	50.6

		[Homo sapiens]		
3891	KRT85	keratin, type II cuticular Hb5 isoform 1 [Homo sapiens]	6.90	55.8
5037	PEBP1	phosphatidylethanolamine-binding protein 1 [Homo sapiens]	26.20	21.0
4332	MNDA	myeloid cell nuclear differentiation antigen [Homo sapiens]	15.97	45.8
10409	BASP1	brain acid soluble protein 1 [Homo sapiens]	29.07	22.7
10095	ARPC1B	actin-related protein 2/3 complex subunit 1B [Homo sapiens]	16.94	40.9
5034	P4HB	protein disulfide-isomerase precursor [Homo sapiens]	10.83	57.1
7317	UBA1	ubiquitin-like modifier-activating enzyme 1 [Homo sapiens]	7.09	117.8
7415	VCP	transitional endoplasmic reticulum ATPase [Homo sapiens]	7.44	89.3
396	ARHGDIA	rho GDP-dissociation inhibitor 1 isoform a [Homo sapiens]	20.59	23.2
3181	HNRNPA2 B1	heterogeneous nuclear ribonucleoproteins A2/B1 isoform A2 [Homo sapiens]	15.84	36.0
4069	LYZ	lysozyme C precursor [Homo sapiens]	22.97	16.5
829	CAPZA1	F-actin-capping protein subunit alpha-1 [Homo sapiens]	20.28	32.9
1890	TYMP	thymidine phosphorylase isoform 1 precursor [Homo sapiens]	14.73	49.9
805	CALM2	calmodulin isoform 2 [Homo sapiens]	16.11	16.8
308	ANXA5	annexin A5 [Homo sapiens]	14.06	35.9
3881	KRT31	keratin, type I cuticular Ha1 [Homo sapiens]	7.21	47.2
6418	SET	protein SET isoform 4 [Homo sapiens]	15.79	31.1
3146	HMGB1	high mobility group protein B1 [Homo sapiens]	13.02	24.9
8288	EPX	eosinophil peroxidase preproprotein [Homo sapiens]	4.62	81.0
307	ANXA4	annexin A4 isoform b [Homo sapiens]	19.06	33.5
7316	UBC	polyubiquitin-C [Homo sapiens]	52.55	77.0
10627	MYL12A	myosin regulatory light chain 12A isoform 1 [Homo sapiens]	28.65	19.8
3047	HBG1	hemoglobin subunit gamma-1 [Homo sapiens]	12.93	16.1
7531	YWHAE	PREDICTED: 14-3-3 protein epsilon	20.42	27.4

		isoform X1 [Homo sapiens]		
1178	CLC	galectin-10 [Homo sapiens]	20.42	16.4
25824	PRDX5	peroxiredoxin-5, mitochondrial isoform a precursor [Homo sapiens]	23.83	22.0
2936	GSR	glutathione reductase, mitochondrial isoform 4 precursor [Homo sapiens]	11.59	47.2
3148	HMGB2	high mobility group protein B2 [Homo sapiens]	11.96	24.0
3178	HNRNPA1	PREDICTED: heterogeneous nuclear ribonucleoprotein A1 isoform X2 [Homo sapiens]	13.56	32.0
3309	HSPA5	78 kDa glucose-regulated protein precursor [Homo sapiens]	9.48	72.3
5696	PSMB8	proteasome subunit beta type-8 isoform E1 precursor [Homo sapiens]	10.29	29.8
1192	CLIC1	chloride intracellular channel protein 1 [Homo sapiens]	21.16	26.9
4688	NCF2	neutrophil cytosol factor 2 isoform 3 [Homo sapiens]	11.01	50.3
6275	S100A4	protein S100-A4 [Homo sapiens]	18.81	11.7
5315	PKM	pyruvate kinase PKM isoform d [Homo sapiens]	8.75	49.9
5273	SERPINB10	PREDICTED: serpin B10 isoform X3 [Homo sapiens]	18.66	30.9
83706	FERMT3	fermitin family homolog 3 short form [Homo sapiens]	9.50	75.4
383	ARG1	arginase-1 isoform 2 [Homo sapiens]	16.77	34.7
3059	HCLS1	hematopoietic lineage cell-specific protein isoform 2 [Homo sapiens]	5.79	49.7
6451	SH3BGRL	SH3 domain-binding glutamic acid-rich-like protein [Homo sapiens]	22.81	12.8
1072	CFL1	cofilin-1 [Homo sapiens]	23.49	18.5
23406	COTL1	coactosin-like protein [Homo sapiens]	21.83	15.9
51411	BIN2	bridging integrator 2 isoform 2 [Homo sapiens]	6.86	59.2
7879	RAB7A	ras-related protein Rab-7a [Homo sapiens]	23.19	23.5
7171	TPM4	tropomyosin alpha-4 chain isoform Tpm4.2cy [Homo sapiens]	10.08	28.5
8125	ANP32A	acidic leucine-rich nuclear phosphoprotein 32 family member A [Homo sapiens]	8.43	28.6
3308	HSPA4	heat shock 70 kDa protein 4 [Homo	5.71	94.3

		sapiens]		
6277	S100A6	protein S100-A6 [Homo sapiens]	24.44	10.2
51571	FAM49B	protein FAM49B [Homo sapiens]	15.74	36.7
3190	HNRNPK	heterogeneous nuclear ribonucleoprotein K isoform c [Homo sapiens]	9.11	48.5
7295	TXN	thioredoxin isoform 2 [Homo sapiens]	15.29	9.4
5777	PTPN6	tyrosine-protein phosphatase non-receptor type 6 isoform 1 [Homo sapiens]	6.89	67.5
7533	YWHAH	14-3-3 protein eta [Homo sapiens]	17.89	28.2
6051	RNPEP	aminopeptidase B isoform a [Homo sapiens]	3.85	72.5
1622	DBI	acyl-CoA-binding protein isoform 3 [Homo sapiens]	50.57	10.0
5479	PPIB	peptidyl-prolyl cis-trans isomerase B precursor [Homo sapiens]	14.81	23.7
826	CAPNS1	PREDICTED: calpain small subunit 1 isoform X2 [Homo sapiens]	17.50	22.9
2992	GYG1	glycogenin-1 isoform 3 [Homo sapiens]	12.19	31.4
6947	TCN1	transcobalamin-1 precursor [Homo sapiens]	9.70	48.2
3958	LGALS3	galectin-3 isoform 1 [Homo sapiens]	10.00	26.1
822	CAPG	macrophage-capping protein isoform 2 [Homo sapiens]	10.81	36.8

Supplemental table2. Sequences of primers

Primer		Sequences	Annealing Temperature
Mouse Lcn2	Sence	5'- GGCCCTGAGTGTCATGTGTC-3'	57 °C
	Antisence	5'- TTCTGATCCAGTAGCGACAGC-3'	
Mouse Il1α	Sence	5'- TGAGTCGGCAAAGAAATCAA -3'	56 °C
	Antisence	5'- AGAGAGATGGTCAATGGCAGA -3'	
Mouse Il1β	Sence	5'- TGCCACCTTTTGACAGTGATG -3'	56 °C
	Antisence	5'- TGATGTGCTGCTGCGAGATT -3'	
Mouse Il6	Sence	5'- CGGAGAGGAGACTTCACAGAG -3'	57 °C
	Antisence	5'- ATTTCCACGATTTCCCAGAG -3'	
Mouse Tnfα	Sence	5'- CCCTCACACTCACAAACCAC -3'	57 °C
	Antisence	5'- ACAAGGTACAACCCATCGGC -3'	
Mouse Il23	Sence	5'-TAATGTGCCCCGTATCCAGT -3'	56 °C
	Antisence	5'-AGGGAGGTGTGAAGTTGCTC -3'	
Mouse Cxcl1	Sence	5'- ACCCAAACCGAAGTCATAGC -3'	57 °C
	Antisence	5'- ACAGGTGCCATCAGAGCAGT -3'	
Mouse Ccl20	Sence	5'- GCAGAAGCAAGCAACTACGAC -3'	57 °C
	Antisence	5'- CTTTGGATCAGCGCACACAG -3'	
Mouse Il36g	Sence	5'- CTGAGCCAATGAAGCCATTC -3'	59 °C
	Antisence	5'- CCAGTCTTGGAGGAGGCAAT -3'	
Mouse Il17A	Sence	5'- CTCAAGGGAAGACGCTGGT -3'	59 °C
	Antisence	5'- AACACCCACAACTCCACCTC -3'	
Mouse TLR4	Sence	5'- GCATGGCTTACACCACCTCTC-3'	59 °C
	Antisence	5'- TGTCTCCACAGCCACCAGAT-3'	

Human IL36R	Sence	5’- CATGTGCCACGCTGGAGTGTC-3’	59 °C
	Antisence	5’- CACAGCCACAGCCACCAAGG-3’	
Human IL-18	Sence	5’- TGTAATCCCAGCCCTTTGG-3’	57 °C
	Antisence	5’- ATTCTCCTGCCTCAGCCTCT3’	
Human CXCL1	Sence	5’- CAGCAGGAGCGTCCGT-3’	57 °C
	Antisence	5’- GTTGGATTTGTCACTGTTCAGC-3’	
Human IL36G	Sence	5’- AGACGCTGATGGTGGAGGAAGG-3’	59 °C
	Antisence	5’- CTGCCTTGCTCAAGAGCCTCTG-3’	
Human LCN2	Sence	5’- CCCAGCCCCACCTCTGA-3’	56 °C
	Antisence	5’- CTTCCCCTGGAATTGGTTGTC-3’	
Human IL-1α	Sence	5’- TGTGACTGCCCAAGATGAAG-3	56 °C
	Antisence	5’- GCCAAGCACACCCAGTAGTC-3’	
Human CXCL8	Sence	5’- CAGCCTTCCTGATTTCTGC-3’	56 °C
	Antisence	5’- AGTTTTCCTTGGGGTCCAG-3’	
Human TNF-α	Sence	5’- AACCTCCTCTCTGCCATCAA-3’	56 °C
	Antisence	5’- CCAAAGTAGACCTGCCCAGA-3’	
Human CCL20	Sence	5’- TTTATTGTGGGCTTCACACG-3’	59 °C
	Antisence	5’- TCACCCAAGTCTGTTTTGGA-3’	
Human IL-6	Sence	5’-CTTCGGTCCAGTTGCCTTCT-3’	59 °C
	Antisence	5’-AGTGCCTCTTTGCTGCTTTC-3’	
Human β-Actin	Sence	5’- GGCTACAGCTTCACCACCAC -3’	57 °C
	Antisence	5’- TGCGCTCAGGAGGAGC -3’	
Human TLR7	Sence	5’- GATTGAAACCTGACCAATTTGCT-3’	56 °C
	Antisence	5’- AAATACGACATCGCCAATCTAAGG -3’	
Human	Sence	5’- CTGCCTTCCTACCCTGTGAG-3’	56°C

TLR9	Antisence	5’- GGATGCGGTTGGAGGACAA-3’	
Human TLR4	Sence	5’- CGAATGGAATGTGCAACACC-3’	57 °C
	Antisence	5’- AAGCACACTGAGGACCGACA-3’	
Human TLR2	Sence	5’- TTCACTCAGGAGCAGCAAGC-3’	57 °C
	Antisence	5’- GTGACATTCCGACACCGAGA-3’	
Human Dectin1	Sence	5’- TGGCAACTGGGCTCTAATCT-3’	57 °C
	Antisence	5’- GCCATGGTACCTCAGTCTGG-3’	
Human MPO	Sence	5’- TTTGACAACCTGCACGATGAC-3’	57 °C
	Antisence	5’- CGGTTGTGCTCCCGAAGTAA-3’	
Human MMP9	Sence	5’- TCATCTTCCAAGGCCAATCC-3’	57 °C
	Antisence	5’- GCAGAAGCCGAAGAGCTTGT-3’	
Human LL37	Sence	5’- GCAGTCACCAGAGGATTGTGAC-3’	59 °C
	Antisence	5’- CACCGCTTCACCAGCCC-3’	
Human cathespin H	Sence	5’- AATAATCACGGCTGCCAAGG-3’	57 °C
	Antisence	5’- ACCATCGCTTCCTCGTCATAG-3’	
Human RNASE2	Sence	5’- CCTCACAACTCCAAGTCCACA-3’	57 °C
	Antisence	5’- TGAACTGGAACCACCGGATA-3’	
Human SLC25A6	Sence	5’- GCTTCAGTGTCTCCGTGCAG-3’	57 °C
	Antisence	5’- CATCCAGCTCACCACGATGT-3’	
Human ATP11B	Sence	5’- CCAGCAACCAGAAGCAGACTT-3’	57 °C
	Antisence	5’- TAATCTGGCTCCACGAAGCA-3’	
Human ATP1B3	Sence	5’- TCCTGGCTGCACTCTTCTCA-3’	57 °C
	Antisence	5’- ATTCCAATGCGGTCACTGGT-3’	
Human CXCL2	Sence	5’- CACTGAACTGCGCTGCCA-3’	57 °C
	Antisence	5’- GCCCATTCTTGAGTGTGGCT-3’	

Human LTF	Sence	5'- ACACTCGGAAGCCAGTGGAC-3'	57 °C
	Antisence	5'-TGGCGGAGAAGATTCCAGAT-3'	
